# Connecting Immune Cell Infiltration to the Multitasking Microglia Response and TNF Receptor 2 Induction in the Multiple Sclerosis Brain

**DOI:** 10.3389/fncel.2020.00190

**Published:** 2020-07-07

**Authors:** Caterina Veroni, Barbara Serafini, Barbara Rosicarelli, Corrado Fagnani, Francesca Aloisi, Cristina Agresti

**Affiliations:** ^1^Department of Neuroscience, Istituto Superiore di Sanità, Rome, Italy; ^2^Centre for Behavioural Sciences and Mental Health, Istituto Superiore di Sanità, Rome, Italy

**Keywords:** multiple sclerosis, brain, laser microdissection, gene expression, microglia, interferon, TNF receptors, remyelination

## Abstract

Signaling from central nervous system (CNS)-infiltrating lymphocytes and macrophages is critical to activate microglia and cause tissue damage in multiple sclerosis (MS). We combined laser microdissection with high-throughput real time RT-PCR to investigate separately the CNS exogenous and endogenous inflammatory components in postmortem brain tissue of progressive MS cases. A previous analysis of immune infiltrates isolated from the white matter (WM) and the meninges revealed predominant expression of genes involved in antiviral and cytotoxic immunity, including IFNγ and TNF. Here, we assessed the expression of 71 genes linked to IFN and TNF signaling and microglia/macrophage activation in the parenchyma surrounding perivascular cuffs at different stages of WM lesion evolution and in gray matter (GM) lesions underlying meningeal infiltrates. WM and GM from non-neurological subjects were used as controls. Transcriptional changes in the WM indicate activation of a classical IFNγ-induced macrophage defense response already in the normal-appearing WM, amplification of detrimental (proinflammatory/pro-oxidant) and protective (anti-inflammatory/anti-oxidant) responses in actively demyelinating WM lesions and persistence of these dual features at the border of chronic active WM lesions. Transcriptional changes in chronic subpial GM lesions indicate skewing toward a proinflammatory microglia phenotype. TNF receptor 2 (TNFR2) mediating TNF neuroprotective functions was one of the genes upregulated in the MS WM. Using immunohistochemistry we show that TNFR2 is highly expressed in activated microglia in the normal-appearing WM, at the border of chronic active WM lesions, and in foamy macrophages in actively demyelinating WM and GM lesions. In lysolecithin-treated mouse cerebellar slices, a model of demyelination and remyelination, TNFR2 RNA and soluble protein increased immediately after toxin-induced demyelination along with transcripts for microglia/macrophage-derived pro- and anti-inflammatory cytokines. TNFR2 and IL10 RNA and soluble TNFR2 protein remained elevated during remyelination. Furthermore, myelin basic protein expression was increased after selective activation of TNFR2 with an agonistic antibody. This study highlights the key role of cytotoxic adaptive immunity in driving detrimental microglia activation and the concomitant healing response. It also shows that TNFR2 is an early marker of microglia activation and promotes myelin synthesis, suggesting that microglial TNFR2 activation can be exploited therapeutically to stimulate CNS repair.

## Introduction

Multiple sclerosis (MS) is the most common chronic inflammatory disease of the central nervous system (CNS) affecting young adults and causing progressive deterioration of motor, sensory, and cognitive functions. To date, it remains unclear whether it is self or non-self-antigens that stimulate the CNS-targeted immune response. Despite extensive search, MS-related autoantigens remain elusive (Willis et al., 2015; Reich et al., 2018). The B-lymphotropic herpesvirus Epstein-Barr virus (EBV) is strongly associated with MS and has been implicated in disease development (Pender and Burrows, 2014). However, the mechanisms linking EBV to MS pathology, including molecular mimicry and immunopathology driven by a persistent EBV infection in the CNS, are debated (Serafini et al., 2019).

Notwithstanding the knowledge gaps regarding MS specific triggers, immune cell recruitment and activation inside the CNS are the most important drivers of resident microglia activation, demyelination and neurodegeneration (Becher et al., 2017; Lassmann, 2019). Blood-derived lymphocytes, including CD4+ T helper cells, CD8+ cytotoxic T cells, B cells and, to a lesser extent, myeloid cells, accumulate predominantly in the connectival spaces of the CNS: the venous perivascular spaces in the white matter (WM) and more rarely in the gray matter (GM) (Lassmann, 2019), and the subarachnoid space of the meninges where ectopic lymphoid-like structures can develop (Serafini et al., 2004). It has been proposed that soluble factors produced by activated lymphocytes and monocytes/macrophages can diffuse into the tissue, inducing microglia activation and CNS tissue damage (Magliozzi et al., 2007, 2010; Machado-Santos et al., 2018). In turn, microglia activation can contribute to both the pathological features of MS lesions and the healing response attenuating inflammation and promoting functional recovery (Butovsky and Weiner, 2018; Li and Barres, 2018).

Previously, we analyzed the expression of a large number of immune-related and EBV genes in laser-cut immune infiltrates from the WM and the meninges of post-mortem MS brain samples (Veroni et al., 2018). We detected EBV genes associated with activation of viral infection and showed an elevated, coordinated expression of genes involved in antiviral immunity, T helper 1 and CD8 T-cell effector functions; these included, among others, genes encoding soluble factors like IFNγ, TNF and lytic enzymes (perforin, granzyme B, metalloproteinases), that are candidate triggers of glia limitans disruption, microglia activation and neural cell injury (Agresti et al., 1996; Agrawal et al., 2006; Sobottka et al., 2009; Denic et al., 2013). During that study, we also microdissected the WM and GM parenchyma contiguous to CNS immune infiltrates. We postulated that studying gene expression changes in these microareas would provide clear-cut information about the impact of peripheral immune cell-derived signals on microglia activation status and lesion evolution.

In this study we used enhanced, multiple target gene real time RT-PCR to assess and compare gene expression in laser-cut MS normal-appearing WM (NAWM), active WM lesions and rim of chronic active WM lesions immediately adjacent to WM perivascular cuffs, chronic subpial GM lesions adjacent to prominent meningeal immune infiltrates, and non-pathological WM and GM. This highly selective and sensitive approach allowed to unravel previously unappreciated changes in the expression of genes linked to IFN and TNF signaling, microglia activation and healing responses in the above mentioned areas. Having found that TNFR2, a receptor implicated in TNF-mediated anti-inflammatory and regenerative processes in the CNS, was upregulated at the transcriptional level in all WM areas analyzed, we performed a detailed study of the distribution and cellular localization of TNFR2 in the MS brain using immunohistochemical techniques. The dynamic regulation of TNFR2 RNA and the therapeutic potential of selective TNFR2 stimulation were explored in an *ex-vivo* mouse model of demyelination/remyelination.

## Materials and Methods

### Human Post-mortem Brain Tissue

All post-mortem brain tissues were obtained from the UK Multiple Sclerosis Tissue Bank at Imperial College London (https://www.imperial.ac.uk/medicine/multiple-sclerosis-and-parkinsons-tissue-bank). A total of 25 snap frozen tissue blocks (2 × 2 × 1 cm) from the superior frontal gyrus, precentral gyrus and middle temporal gyrus of 16 cases of progressive MS (1–3 blocks/case) and 5 brain tissue blocks from 5 non-neurological control cases were selected for this study (Table 1). Based on the available clinical histories, all patients had developed secondary progressive MS, except MS121 and MS234 who had developed relapsing progressive MS, and MS176 case who developed primary progressive MS. All patients were wheelchair- or bed-bound at the time of death [Expanded Disability Status Scale (EDSS) > 7.5]. For some patients, information was also available on immunotherapies received during the relapsing-remitting phase of the disease, whereas no treatment was reported during the progressive phase of MS. Use of human tissue for research purposes was approved by the Ethics Committee of Istituto Superiore di Sanità (CE 12/356). Brain specimens were analyzed and classified by histopathological methods, as previously described (Serafini et al., 2006, 2010). Demographic, clinical and neuropathological data, and post-mortem delay intervals are summarized in Table 1.

**Table 1:** Demographic, clinical and neuropathological characteristics of the MS and control cases analyzed.

**MS case/n° brain blocks**	**Sex/Age at death**	**Disease duration (years)**	**Cause of death**	**Immunotherapy**	**Post-mortem delay (hrs)**	**Use**	**Number and type of WM/GM areas used for IHC^a^ and IF^b^**	**Number and type of laser-cut WM/GM areas used for gene expression analysis**
MS56/1	M/63	39	Pneumonia, MS	Age 44: ACTH	11	IHC, IF	2 chronic active WML^c^; 1 chronic active GML^d^	N.P.^e^
MS79/2	F/49	24	Bronchopneumonia, MS	Age 28: ACTH, azathioprine for 1 year Until age 35, courses of methylprednisolone	7	IHC, IF LCM^g^	1 chronic active WML; 1 NAWM; 1 chronic active GML; 1 chronic inactive GML	1 NAWM^f^; 1 active and 1 chronic active WML; 1 chronic active GML
MS92/3	F/37	17	MS	Age 21: ACTH	26	LCM	N.P.	6 NAWM; 2 chronic inactive GML
MS100/1	M/46	8	Pneumonia, MS	Age 38: prednisolone Age 39: methylprednisolone Age 41: cyclophosphamide	7	IHC, IF	1 NAWM; 1 chronic active GML	N.P.
MS121/2	F/49	14	Pneumonia, MS	Age 46: methylprednisolone	24	IHC, IF LCM	1 NAWM; 1 active WML; 1 chronic inactive GML	4 NAWM; 2 active and 1 chronic active WML
MS154/2	F/35	11	Pneumonia, MS	Not reported	12	IHC, IF	1 active WML; 1 chronic active WML	N.P.
MS160/2	F/44	15	Aspiration pneumonia, MS	Not reported	18	IHC, IF LCM	1 NAWM; 1 chronic active WML; 2 active GML 2 chronic active GML; 1 chronic inactive GML	2 chronic inactive GML
MS163/1	F/45	6	Sepsis, MS	Not reported	28	IHC, IF	1 NAWM	N.P.
MS176/1	M/37	27	Intestinal obstruction, MS	Age 21: ACTH Age 22: methylprednisolone, cyclophosphamide	12	IHC, IF LCM	1 active WML	3 active WML; 1 chronic active WML; 1 chronic inactive GML
MS180/2	F/44	18	MS	Not reported	7	IHC, IF LCM	1 active WML; 1 chronic active GML	1 NAWM; 5 active and 3 chronic active WML; 3 chronic active and 2 chronic inactive GML
MS200/1	F/44	19	Sepsis, MS	Age 39: azathioprine for 11 months	20	IHC, IF	1 chronic active WML; 1 chronic active GML; 1 chronic inactive GML	N.P.
MS234/2	F/ 39	15	Pneumonia, MS	Not reported	15	LCM	N.P.	1 chronic active GML; 5 chronic inactive GML
MS 352/1	M/43	18	MS	Age 32: methylprednisolone Age 33: Campath-1H	26	LCM	N.P.	1 chronic inactive GML
MS356/1	F/45	16	MS	Not reported	10	IHC, IF	1 chronic active WML; 1 chronic active GML	N.P.
MS402/1	M/46	20	Bronchopneumonia, MS	Not reported	12	LCM	N.P.	1 active WML; 1 chronic inactive GML
MS407/2	F/44	19	Septicemia, pneumonia, MS	Age 32: interferon β-1a for 10 years	22	LCM	N.P.	2 NAWM; 1 active and 1 chronic active WML; 1 chronic active GML; 4 chronic inactive GML
**Control cases**	**Sex/Age at death**		**Cause of death**	**Therapy**	**Post-mortem delay (hrs)**	**Use**	**Main neuropathological features**	**Number and type of laser-cut WM/GM areas used for gene expression analysis**
C14/1	M/64		Cardiac failure	Not reported	18	IHC LCM	Normal/History of transient ischaemic attacks	1 WM; 1 GM
C25/1	M/35		Carcinoma of the tongue	Not reported	22	IHC LCM	Normal	1 WM; 2 GM
C28/1	F/60		Ovarian cancer	Not reported	13	LCM	Scarse inflammatory infiltrates	1 WM; 1 GM
C30/1	M/75		Cerebrovascular accident	Not reported	17	IHC, IF LCM	Aging, Alzheimer's disease-like pathology	1 WM; 1 GM
C45/1	M/77		Cardio pulmonary degeneration	Not reported	22	IHC LCM	Aging	1 WM; 1 GM

*MS cases had developed secondary progressive MS, except MS121 and MS234, who developed relapsing progressive MS, and MS176 who developed primary progressive MS*.

a *IHC, Immunohistochemistry*;

b *IF, Immunofluorescence*;

c *WML, white matter lesion*;

d *GML, gray matter lesion*;

e *N.P., not performed*;

f *NAWM, normal-appearing white matter*;

g *LCM, laser capture microdissection*.

### Laser Capture Microdissection

**Figure 1:**
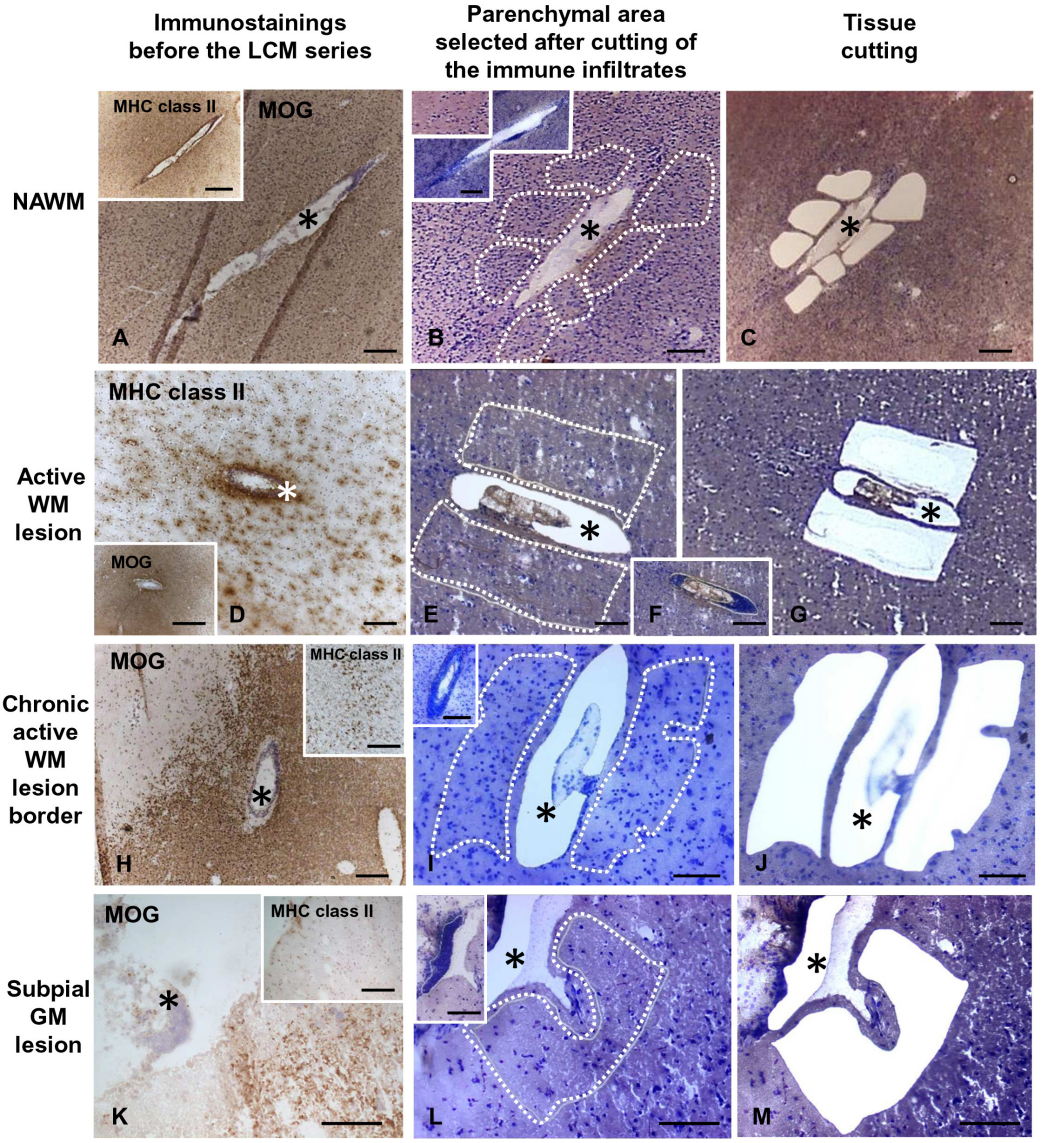
Immunohistochemical characterization and laser capture microdissection of the white matter and gray matter parenchyma adjacent to perivascular cuffs and meningeal infiltrates in the MS brain. Brain sections were immunostained with anti-MOG and anti-MHC class II antigen (anti-HLA-DP, DQ, DR) antibodies and counterstained with hematoxylin to assess the degree of demyelination and inflammation and identify the relevant brain areas (**A,D,H,K** and insets). Following rapid nuclear staining of serial sections, immune infiltrates (**F**, and insets in **B,I,L**) and the adjacent NAWM **(B,C)**, active WM lesions **(E,G)**, border of chronic active WM lesions **(I,J)**, or subpial GM lesions **(L,M)** were cut consecutively and collected separately. The asterisks mark perivascular **(A–J)** and meningeal **(K–M)** immune infiltrates before and after microdissection. The stainings shown in this figure were performed in brain samples of three MS cases. Bars = 500 μm in the insets in **A,D,H**; 200 μm in **A–D,F,H–K**, insets in **B,I,K**; 100 μm in **E,G,L,M** and inset in **L**.

Snap frozen brain tissue blocks from five control cases [median post-mortem delay (PMD) = 18 h] and 10 MS cases (median PMD = 13.5 h) with RNA Integrity Number (RIN) values ≥ 6 (Veroni et al., 2018) were used for laser capture microdissection (LCM) and subsequent gene expression analysis (Table 1). RNA was extracted from each tissue block using RNeasy mini kit (Qiagen, Valencia, CA) and RNA quality was evaluated using Agilent 2100 Bioanalyzer. Brain sections (10-μm thick) were stained with hematoxylin and eosin (H&E) to localize WM perivascular and meningeal immune infiltrates and with antibodies specific for myelin oligodendrocyte glycoprotein (MOG) and MHC class II molecules (HLA-DP, DQ, DR) to assess the extent of demyelination and inflammatory activity, respectively, as described (Serafini et al., 2006, 2010). Eight-10 serial brain sections adjacent to those used for neuropathological characterization were cut with a cryostat in RNAse-free conditions, mounted on membrane-coated microscopy nuclease and nucleic acid free slides (MMI AG, Glattbrugg, Switzerland), and subjected to rapid nuclear staining and dehydration procedures (Arcturus Histo Gene Staining Solution, Life Technologies, Grand Island, NY). Sections were air-dried for 1 h and LCM was performed using a laser microdissector SL Cut (MMI AG) equipped with a UV-Cut SL Microtest software and a Nikon Eclipse TE2000-S microscope. CNS immune infiltrates and the adjacent neural parenchyma were collected consecutively (Figure 1). The procedure was performed in RNase-free conditions to avoid RNA degradation. The WM and GM parenchyma contiguous to WM perivascular cuffs and sparse or lymphoid-like meningeal immune infiltrates, respectively, included: NAWM with activated microglia and apparently normal myelin; active and demyelinating WM lesions, characterized by the presence of MOG-containing macrophages throughout the lesion area; the hypercellular border of chronic active WM lesions comprising macrophages and activated microglia; subpial chronic active and inactive GM lesions characterized by the presence and absence of a rim of MHC class II+ microglia surrounding the demyelinated area, respectively.

Each WM and GM sample was isolated from 3 to 5 serial sections and the fragments were collected in a single cap. The pooled tissue fragments of each series were incubated immediately in 50 μl of RNA stabilizing extraction buffer (PicoPure RNA isolation kit, Arcturus, Life Technologies) at 42°C for 30 min and centrifuged at 800 × g for 2 min. Lysates were stored at −80°C until use. The area of the pooled microdissected parenchymal areas ranged from 162.000 to 1.168.000 μm^2^ (median value = 645.500 μm^2^) for the WM and from 194.000 to 1.000.000 μm^2^ (median value = 582.000 μm^2^) for the GM. The final sample cohort included 35 WM and 24 GM samples from 10 MS cases, and 5 WM and 6 GM samples from five control cases (Table 1).

### High-Throughput Real Time Reverse Transcription PCR

Total RNA was extracted from the laser-cut WM and GM samples using Picopure RNA isolation kit (Arcturus, Life Technologies). The low amount of RNA extracted from laser-cut samples did not allow to check for RNA quality and quantity. The whole volume of purified RNA was reverse transcribed immediately after extraction using the High Capacity Reverse Transcription kit. RNase inhibitor (Life Technologies) was added to minimize RNA degradation. cDNA was preamplified for a total of 79 cellular transcripts (including the housekeeping gene GAPDH) and 2 EBV genes (EBER1 and LMP1) using a pool of TaqMan Gene Expression Assays (Supplementary Table 1) and the TaqMan PreAmp Master Mix (Life Technologies). The preamplification reaction was carried out at the following thermal conditions: 50°C for 2 min and 95°C for 10 min, followed by 95°C for 15 s and 1 min at 60°C for 14 cycles. The final product was diluted 1:5 in TE buffer and analyzed using the high-throughput BioMark real time PCR nanofluidic platform (Fluidigm, San Francisco, CA). For each sample, a mix composed by 3 μl of TaqMan Gene Expression Master Mix (Life Technologies), 0.3 μl of 20 × sample loading reagent (Fluidigm), and 2.7 μl of preamplified sample was prepared. For each assay, 3 μl of the 2 × assay loading reagent (Fluidigm) were mixed with 3 μl of each TaqMan Gene Expression assay used in the preamplification step (Life Technologies). After BioMark 48.48 Dynamic Array IFC chip (Fluidigm) priming, both the sample and assay mix were loaded in duplicate into the chip inlets (to have four independent reactions for each sample/assay pair) using the IFC controller MX (Fluidigm). A no-template preamplification control was always included in each chip. The loaded 48.48 DA IFC chip was finally processed using the high-throughput RT-PCR instrument (BioMark; Fluidigm) at the following thermal conditions: 94°C for 2 min, followed by 40 cycles of 94°C for 15 s, and 60°C for 60 s. Data were acquired and analyzed using the Biomark Real-Time PCR Analysis Software (Fluidigm). Gene expression levels are expressed as 2^−ΔCt^ value relative to the endogenous GAPDH mRNA.

### Immunohistochemistry and Immunofluorescence Staining of MS and Control Brain Samples

Snap frozen brain tissue blocks from 11 MS cases and four control cases were used for immunohistochemical and immunofluorescence experiments (Table 1). Ten-μm-thick sections were cut and stored at −80°C. Acetone- or methanol-fixed (for MOG staining) sections were rehydrated with phosphate buffered saline (PBS), pre-incubated with 10% normal serum (NS) and immunostained overnight at 4°C with the following primary antibodies diluted in PBS containing 2% bovine serum albumin (BSA): anti-MOG mouse monoclonal antibody (mAb) (1:1,000, clone 2858; Novus Biologicals, Oxford, UK), anti-HLA-DP, DQ, DR antigen mouse mAb (1:50, clone CR3/43; DakoCytomation, Glostrup, Denmark), anti-CD68 mouse mAb (1:50; clone KP1, DakoCytomation), anti-TNFRSF1B (TNFR2) rabbit polyclonal Ab (1:50; Sigma-Aldrich, St. Louis, MO). After washing with PBS, sections were incubated consecutively with 0.1% H_2_O_2_ in PBS for 20 min in the dark to eliminate endogenous peroxidase activity, biotinylated secondary Abs (rabbit anti-mouse or goat anti-rabbit, final concentration 3 μg/ml; Jackson ImmunoResearch Laboratories, West Grove, PA) diluted in PBS containing 5% NS for 1 h, and avidin-biotin horseradish peroxidase complex (ABC Vectastain Elite kit, Vector Laboratories, Burlingame, CA) for 45 min at room temperature (RT). Staining reactions were performed with 3,3′-diaminobenzidine (Sigma-Aldrich) or 3-amino-9-ethylcarbazole (DakoCytomation) as chromogen. Negative controls included the use of mouse IgG1 isotype control (control for mAbs) or pre-immune rabbit serum (control for polyclonal Abs) (both from Jackson ImmunoResearch Laboratories), omission of primary Abs (control for all stainings), and pre-adsorption of anti-TNFR2 Ab with TNFRSF1B PrEST Antigen (APrEST86776, Sigma-Aldrich) following the manufacturer's instructions. Sections were counterstained with hematoxylin and analyzed with an Axiophot microscope (Carl Zeiss, Jena, Germany) equipped with a digital camera (Axiocam MRC); images were acquired using Axiovision 4 AC software.

For double indirect immunofluorescence, brain sections were incubated overnight at 4°C with a mixture of anti-TNFR2 rabbit polyclonal Ab and one of the following mAbs: anti-CD68, anti-2′,3′-cyclic nucleotide-3′-phosphodiesterase (CNPase) (1:50; clone 11-5B, GeneTex Inc., Irvine, CA) or anti-glial fibrillary acidic protein (GFAP) (1:500, clone GA-5, BioGenex, San Ramon, CA) diluted in PBS containing 1% BSA. Sections were washed three times in PBS plus Triton X-100 0.02%, and then incubated for 1 h at RT with Alexa Fluor 488-conjugated goat anti-mouse IgG (1:300, Invitrogen, Eugene, OR) and biotinylated goat anti-rabbit IgG followed by tetramethyl rhodamine isothiocyanate (TRITC)-conjugated streptavidin (dilution 1:150). Sections were sealed in ProLong Gold antifade reagent with 4′,6′-diamidino-2-phenylindole (DAPI; Invitrogen) and images were acquired and analyzed with a Leica fluorescence microscope (DM-4000B, Leica Microsystems, Wetzlar, Germany). Cells co-expressing TNFR2 and CD68, GFAP or CNPase were counted in 5–10 random fields within the areas of interest using a 20× objective by two independent investigators (B.S. and B.R.). Cell counts were performed in brain sections from 7 MS cases. Data are expressed as percentages of double positive cells in the total TNFR2+, CD68+, GFAP+, or CNPase+ cell populations; ranges and median values are presented.

### Animals

CD1 Swiss mice were purchased from Harlan Laboratories (San Pietro Al Natisone, Udine, Italy). The study was approved by the National Center for Animal Research and Welfare of the Istituto Superiore di Sanità and by the Italian Ministry of Health (Authorization 271/SSA/2010).

### Organotypic Mouse Cerebellar Slice Cultures

For demyelination/remyelination experiments, 350 μm-thick slices were prepared from the cerebellum of postnatal day 10 (P10) mice, as described (Eleuteri et al., 2017). P10 cerebellum was used because at this developmental stage Purkinje cells have a good survival rate in culture (Dusart et al., 1997). Slices were cultured in 1 ml of medium (50% basal medium with Earle's salts, 25% Hanks' balanced salt solution, 25% horse serum, and 5 mg/ml glucose) at 35°C in a 95% air-5% CO_2_ humidified atmosphere, replacing fresh medium after 1 day and then every 2–3 days. After 1 week, when significant *in vitro* myelination occurred, 0.5 mg/ml of lysolecithin (Sigma-Aldrich) was added to the culture medium for 16 h to induce demyelination (Birgbauer et al., 2004). For gene expression analysis, cerebellar slices were collected at 0, 2, and 4 days after toxin removal and used for gene expression analysis. To investigate the effect of TNFR2 selective activation, slices were treated immediately after lysolecithin removal with agonistic anti-TNFR2 rat mAb (2 μg/ml; clone HM102, Hycult Biotechnology, Uden, NL) or control isotype rat IgG2a (2 μg/ml; BD Pharmingen, Franklin Lakes, NJ) and collected after 4 days for gene expression analysis and after 7 days for double immunofluorescence stainings.

### Real Time RT-PCR in Mouse Cerebellar Slices

Cerebellar slices were washed in PBS, removed from the inserts and centrifuged at 1,000 × g for 10 min at 4°C. Two cerebellar slices were pooled for each experimental point. Supernatants were discarded and total RNA was isolated from the pellets using RNeasy Micro kit (QIAGEN) and measured by Nanodrop (Thermo Fisher Scientific, Waltham, MA). Five-hundred nanograms of RNA were reverse transcribed using the High Capacity Reverse Transcription kit (Life Technologies); cDNA was amplified on ABI PRISM 7500 Real-Time PCR instrument, using the TaqMan Gene Expression Master Mix (Life Technologies) and inventoried FAM-labeled gene expression assays (Life Technologies) for the following target genes: platelet derived growth factor receptor α (PDGFRα; assay ID Mm00440701_m1), UDP glycosyltransferase 8 (UGT8; assay ID Mm00495930_m1), myelin basic protein (MBP; assay ID Mm01266402_m1), TNFR1 (Mm00441889_m1), TNFR2 (Mm00441875_m1), CD11b (Mm00434455_m1), TNF (Mm00443258_m1), IL1β (Mm00434228_m1), and IL10 (Mm01288386_m1). Each sample was analyzed in triplicate. VIC dye-labeled GAPDH was used as housekeeping gene (Veroni et al., 2010) for all target genes. Gene expression levels were calculated using the formula 2^−ΔCt^ or 2^−ΔΔCt^, where ΔCt is the difference in cycle threshold (Ct) between target mRNA and GAPDH mRNA and ΔΔCt is the difference between ΔCt of treated slices and ΔCt of untreated slices.

### Quantification of Soluble Mouse TNFR1 and TNFR2

Cerebellar slices were cultured for 7 days and then incubated without or with lysolecithin (0.5 mg/ml) for 16 h. Toxin-containing medium was removed and replaced with fresh medium. After 8, 24 and 48 h, 60 μl of medium were collected from lysolecithin-treated and untreated slices, centrifuged and stored at −20°C until use. TNFR1 and TNFR2 secreted in the culture supernatants were quantified using ELISA kits specific for mouse TNFR1 and TNFR2 (R&D Systems, Minneapolis, MN), respectively.

### Immunofluorescence Staining of Mouse Cerebellar Slices

Cerebellar slices were analyzed using double immunofluorescence staining for MBP and neurofilament heavy chain (NFH), as described (Eleuteri et al., 2017). Slices were fixed in 4% paraformaldehyde for 50 min at RT and then removed from the inserts. Free floating slices were incubated for 3 h with PBS, 10% donkey serum, 1% bovine serum albumin and 0.2% Triton X-100 at RT, and then with anti-MBP mouse mAb (clone SMI 99, 1:1,000; Covance Research Products, Denver, PA) and anti-NFH rabbit polyclonal Ab (1:500, AbD Serotec, Oxford, UK) overnight at 4°C. Incubation with secondary Abs was carried out for 1 h at RT using Alexa Fluor 488-conjugated donkey anti-mouse IgG (Invitrogen) and Cy3-coniugated donkey anti-rabbit IgG (1:400, Jackson ImmunoResearch Laboratories). Slices were mounted on slides in Vectashield (Vector Laboratories) and images were acquired with a LSM 5 Pascal Laser Scanning Microscope (Carl Zeiss).

To evaluate the effect of TNFR2 agonistic mAb on myelin protein expression in remyelinating cerebellar slices, we obtained stacks of photographs of MBP and NFH immunofluorescence stainings at 2 μm intervals at 20X magnification using the LSM 5 Pascal Laser Scanning Microscope. Five stacks for each experimental point were analyzed, quantifying the mean fluorescence intensity of each fluorochrome. The index of MBP expression was calculated as the ratio between MBP and NFH fluorescence intensities in three separate experiments.

### Statistical Analysis

Univariate and multivariate statistical approaches were used to test the study hypotheses. The analyses were mainly based on nonparametric statistics to account for departure from normality in the relatively small sample investigated. Between-group comparisons for continuous variables were performed using Mann-Whitney test and Kruskal-Wallis test with *post-hoc* Dunn's test in their cluster-adjusted version to account for multiple (i.e., correlated) measures within cases. The problem of multiple testing has been addressed using the Bonferroni correction in order to reduce the chance of type I errors. Continuous and categorical variables were summarized as means and standard deviations or medians and interquartile ranges, and percentages, respectively. In order to unravel coordinated gene expression patterns, data from WM and GM areas of MS cases were examined from a multivariate perspective by applying factor analysis. Given the relatively low number of observations, the set of variables (i.e., genes) to be used for this analysis was reduced by the following procedure: (i) rarely expressed genes were excluded; (ii) only genes with moderate to high Spearman correlation with at least two other genes were considered (*n* = 40). Exploratory factor analysis (EFA) was carried out through the principal factor extraction method with orthogonal varimax rotation. The decision on the optimal number of factors to be retained was guided by multiple criteria: eigenvalues exceeding 1.0, visual inspection of the scree plot, and explained variance. In interpreting the factor solution, only those original variables with factor loadings higher than 0.5 in absolute value were considered. The scores of each subject in each of the EFA-derived empirical factors were included as continuous variables in subsequent analyses. The five-factor solution obtained from WM data was exploited to derive the number of independent statistical comparisons to be used for a *post-hoc* Bonferroni-like correction for multiple testing in the comparisons between control and MS WM samples (corrected *p*-value threshold of 0.01 (i.e., 0.05/5)]. Receiver operating characteristic (ROC) curve analysis and related statistics—i.e., area under the curve (AUC) and its 95% confidence interval (CI)—were used to evaluate the power of empirical factors in discriminating groups. All analyses were performed separately for the WM and GM using the Stata software (Release 16).

In the experiments with mouse cerebellar slices, comparisons for repeated measures over time were performed using two-way ANOVA with *post-hoc* Bonferroni correction; comparisons between two groups were performed using paired Student's *t*-test (*p* < 0.05).

## Results

### Laser Capture Microdissection of White Matter and Gray Matter Parenchyma From MS and Control Brains

Serial brain sections for LCM and subsequent RNA analysis were cut from 18 tissue blocks of 10 progressive MS cases (Table 1) that were selected for presence of substantial immune infiltrates in the WM and meninges and for good RNA quality (RIN ≥ 6, as detailed in Veroni et al., 2018). For each series, the immune infiltrates were microdissected first, followed by the nearby neural parenchyma (Figure 1). The WM areas surrounding medium to large size perivascular cuffs included: (i) NAWM (*n* = 14) characterized by apparently normal myelin and activated microglia (Figures 1A–C), possibly evolving into actively demyelinating lesions; (ii) active WM lesions characterized by myelin breakdown and presence of foamy macrophages throughout the lesion area (*n* = 13) (Figures 1D–G); these lesions usually evolve into chronic active lesions; (iii) the edge of chronic active WM lesions, also denominated mixed active/inactive WM lesions (Kuhlmann et al., 2017), defined as the rim of activated microglia/macrophages surrounding the demyelinated, inactive lesion core (*n* = 8) (Figures 1H–J). The microdissected chronic active and inactive subpial GM lesions (*n* = 24) were adjacent to large diffuse or lymphoid-like meningeal infiltrates (Figures 1K–M). Non-infiltrated WM (*n* = 5) and cortical GM (*n* = 6) areas were microdissected from brain tissue blocks of non-neurological control cases. Age at death, female:male ratio and postmortem interval were not significantly different between MS and control cases (*p* = 0.075, *p* = 0.067, and *p* = 0.78, respectively).

**Figure 2:**
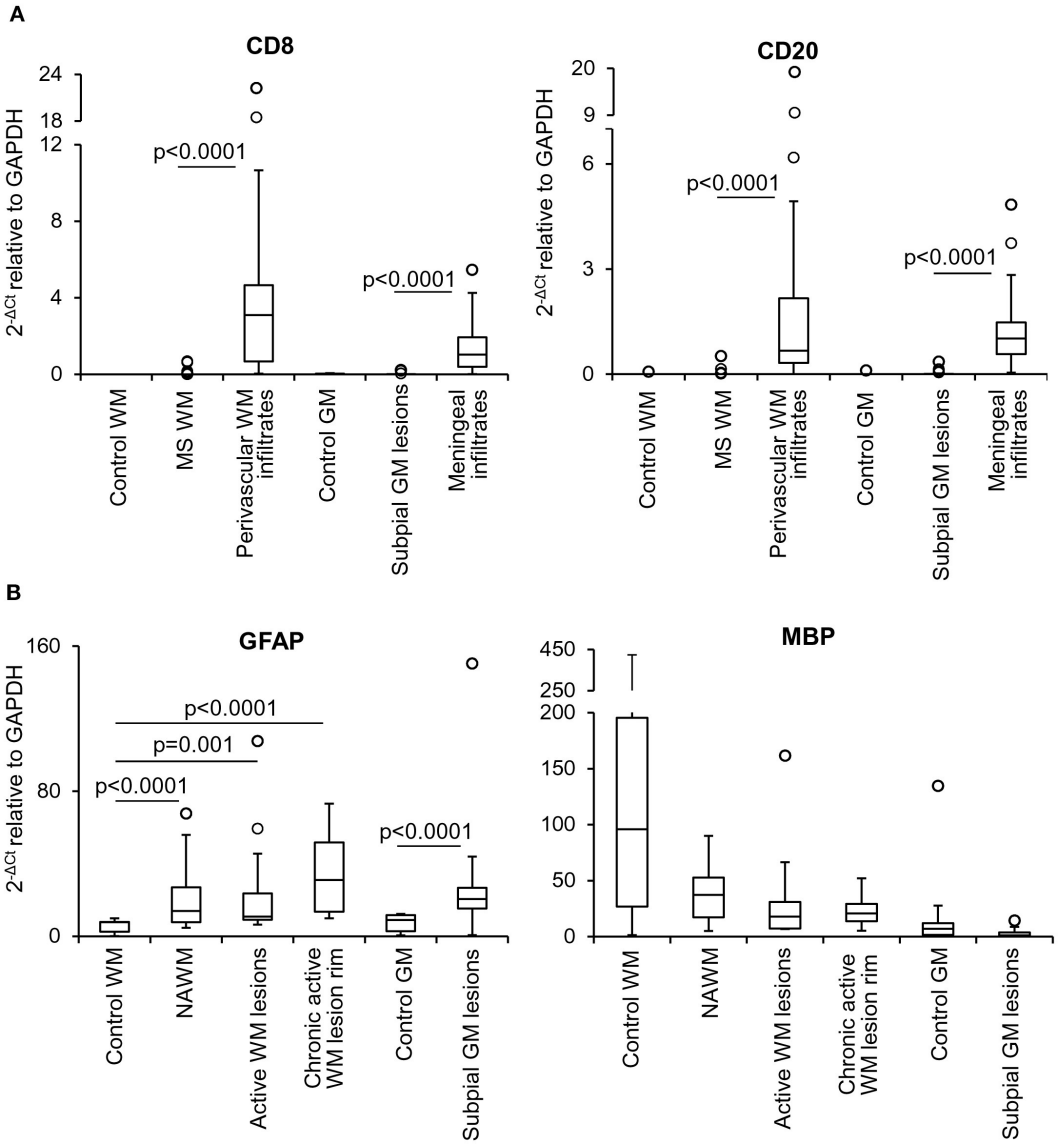
Levels of lymphocyte, astrocyte and myelin transcripts in laser-cut white matter and gray matter samples from control and MS brains. RNA was extracted and reverse transcribed from microdissected brain samples and expression of the indicated genes was analyzed using preamplification real time RT-PCR. **(A)** Levels of genes encoding T cell (CD8) and B cell (CD20) specific markers were assessed in samples microdissected from control WM (*n* = 5), control GM (*n* = 6), MS WM parenchyma (*n* = 35; pooled data from NAWM, active WM lesions and chronic active WM lesion rim), subpial GM lesions (*n* = 24), WM perivascular (*n* = 35), and meningeal (*n* = 24) infiltrates. Data obtained in the MS WM or GM were compared with those obtained in the control WM or GM and in the contiguous WM perivascular or meningeal immune infiltrates, respectively, using the Mann-Whitney test. Statistically significant differences are shown (p < 0.025, after Bonferroni correction). **(B)** Gene expression levels of astrocyte (GFAP) and myelin (MBP) specific markers in control WM (*n* = 5) and GM (*n* = 6) samples were compared with those obtained in NAWM (*n* = 14), actively demyelinating WM lesions (*n* = 13), rim of chronic active WM lesions (*n* = 8) and subpial chronic GM lesions (n = 24), respectively. Statistically significant differences are shown (*p* < 0.017, after Bonferroni correction, for WM comparisons; *p* < 0.05 for GM comparison). The lines inside the boxes represent the median value; boxes extend from the 25th to the 75th percentile, covering the interquartile range (IQR), and whiskers extend from the 25th percentile – 1.5 IQR to the 75th percentile + 1.5 IQR. Outliers outside the whiskers are represented by individual marks.

Multiple target gene, preamplification real time RT-PCR was used to investigate the transcriptional profile of the microdissected MS and control WM and GM samples. Besides ensuring a high degree of specificity, this approach has the double advantage of improving detection of low frequency transcripts and enabling analysis of a large number of transcripts even with very low amounts of starting RNA (Veroni et al., 2018). Among the cellular genes analyzed (*n* = 78), some were selected to check for peripheral blood cell contamination and pathologic status of the microdissected areas. A preliminary analysis showed that transcripts present in specific immune cell subsets, like T cells (CD8, Fas ligand, IFNγ), B cells (CD20) and plasma cells (CD138) were either undetectable (Fas ligand, IFNγ, CD138) (not shown) or present at very low levels in 20–30% of the MS WM and GM samples (CD8, CD20). As shown in Figure 2A, CD8 and CD20 gene expression levels in MS brain parenchymal samples did not differ from those detected in control brain parenchyma and were negligible compared to MS immune infiltrates. These data indicate that the microdissected MS brain parenchyma included no or minimal amounts of blood-derived inflammatory cells. The EBV latent transcripts EBER1 and LMP1 were not detected in any of the microdissected MS WM and GM samples (data not shown), a finding consistent with EBV infection being restricted to B cells accumulating in the MS brain connectival spaces (Serafini et al., 2007, 2010; Angelini et al., 2013; Veroni et al., 2018). Differences in GFAP RNA levels between MS and control samples were consistent with marked astrocyte activation in all MS brain parenchymal areas analyzed (Figure 2B). Although not statistically significant, a decrease in MBP gene expression was found in WM and GM lesions as well as in the NAWM relatively to control samples (Figure 2B), in line with previous observations (Koning et al., 2007).

### Gene Expression Analysis of White Matter and Gray Matter Parenchyma From MS and Control Brains

The genes selected to evaluate the local inflammatory response in the microdissected MS brain parenchymal areas are listed in Table 2 and include: genes that are primarily or exclusively expressed in microglia and play a role in signaling to microglia/macrophages (Zrzavy et al., 2017; Butovsky and Weiner, 2018); transcription factors regulating microglia/macrophage differentiation and activation (Gunthner and Anders, 2013; Butovsky and Weiner, 2018); genes involved in IFN (Langlais et al., 2016; Platanitis and Decker, 2018) and TNF (Brenner et al., 2015) signaling, inflammation and anti-microbial response [including genes encoding inflammasome components and guanylate binding proteins (GBPs) (Kim et al., 2016; Tretina et al., 2019)], antigen presentation (Aloisi, 2001), phagocytosis (PrabhuDas et al., 2017), nitric oxide and oxygen radical production (Schuh et al., 2014; Vilhardt et al., 2017), protection from oxidative stress (Vilhardt et al., 2017), extracellular matrix degradation (Huang et al., 2012) and pathogen recognition (Hanke and Kielian, 2011); genes encoding cytokines/cytokine receptors and chemokines/chemokine receptors expressed by microglia/macrophages or regulating microglia/macrophage functions (Aloisi, 2001; Arango Duque and Descoteaux, 2014; Ruytinx et al., 2018; Viola et al., 2019). Many of the selected genes are inducible by IFNγ (Langlais et al., 2016), which is a major trigger of microglia/macrophage effector functions involved in antimicrobial defenses and is produced by MS brain-infiltrating immune cells (Serafini et al., 2007; Magliozzi et al., 2018; Veroni et al., 2018).

**Table 2:** Genes analyzed in the white matter and gray matter parenchyma from control and MS brains.

Microglia-specific molecules	TMEM119, P2RY12
Microglia/macrophage signaling molecules	TREM2, CSF1R
Transcription factors	RUNX1, IRF1, IRF4, IRF8
IFN-related genes	IFNβ, IFNγR1, JAK2, STAT1, STAT2, IFI6, IFI16, IFIT1, MXA, OAS1, OAS2
Antigen presentation, T-cell and macrophage interaction	Cathepsin S, CIITA, RFX5, HLA-DRA, CD86, CD40
Scavenger receptors/phagocytosis	CD68, MSR1, MRC1, CD163, CXCL16
Inflammatory response	GBP1, GBP2, GBP4, GBP5, NLRP3, Caspase 1, COX2
Cytokines/receptors	TNF, TNFR1, TNFR2, IL1α, IL1β, IL6, IL10, IL16, IL18, BAFF, TGFβ, GMCSF, MCSF/CSF1, SPP1
Chemokines/receptors	CCL2, CCL4, CCL5, CXCL10, CCR1, CCR2, CX3CR1
Pro-oxidant molecules	iNOS, CYBB, CYBA
Anti-oxidant molecules	NRF2, GPX1, HMOX1
Metalloproteinases	MMP1, MMP2, MMP9
Pattern recognition receptors	TLR2, TLR3, TLR7, TLR9

The expression level and frequency of all the analyzed immune-related genes in the total laser-cut sample cohort are summarized in Supplementary Table 2; in this table fold-changes in gene expression values between control and MS brain samples are also shown. Among 71 genes, only TNF and GMCSF were undetectable in all samples analyzed. Gene expression levels in the different perivascular WM areas (NAWM, active WM lesions, chronic active WM lesion rim) and in subpial GM lesions were compared with those in control WM and GM samples, respectively, using the Mann-Whitney test. We identified numerous differentially expressed genes that were upregulated in MS brain-derived samples compared to control brain samples (Table 3); no downregulated genes were found. Box plots depicting the distribution of gene expression values in each sample group are shown in Supplementary Figure 1; only TNFR1 and TNFR2 gene expression data are plotted in **Figure 4**.

**Table 3:** Genes upregulated in the MS white matter and gray matter parenchyma.

**Gene**	**NAWM vs. control WM**	**Active WM lesion vs. control WM**	**Chronic active WM lesion rim vs. control WM**	**Subpial GM lesions vs. control GM**
TMEM119	n.s.	n.s.	n.s.	<0.0001
CSF1R	<0.0001	<0.0001	n.s.	n.s.
TREM2	<0.0001	<0.0001	<0.0001	n.s.
RUNX1	n.s.	0.003	0.024	n.s.
IRF1	0.011	0.001	n.s.	n.s.
IRF4	0.001	<0.0001	0.005	n.s.
IRF8	<0.0001	<0.0001	<0.0001	n.s.
IFNβ	n.s.	0.001	n.s.	<0.0001
STAT1	n.s.	<0.0001	n.s.	n.s.
STAT2	n.s.	0.022	n.s.	n.s.
IFI6	n.s.	0.011	n.s.	n.s.
IFI16	0.048	<0.0001	<0.0001	<0.0001
MxA	0.023	<0.0001	n.s.	n.s.
OAS1	n.s.	0.006	n.s.	n.s.
Cathepsin S	<0.0001	<0.0001	<0.0001	<0.0001
CIITA	0.042	0.025	n.s.	0.004
RFX5	0.001	<0.0001	n.s.	n.s.
HLA-DRA	<0.0001	<0.0001	<0.0001	<0.0001
CD86	<0.0001	<0.0001	0.003	n.s.
CD40	<0.0001	<0.0001	0.003	0.02
CD68	<0.0001	<0.0001	<0.0001	0.045
MSR1	<0.0001	<0.0001	<0.0001	n.s.
CD163	n.s.	0.044	n.s.	0.003
Caspase 1	<0.0001	<0.0001	<0.0001	<0.0001
IL1β	n.s.	n.s.	n.s.	<0.0001
IL10	<0.0001	<0.0001	n.s.	n.s.
IL16	0.045	0.005	n.s.	n.s.
IL18	<0.0001	n.s.	n.s.	0.01
BAFF	n.s.	<0.0001	n.s.	0.003
TGFβ1	0.03	<0.0001	<0.0001	n.s.
TNFR1	0.016	<0.0001	n.s.	0.004
TNFR2	<0.0001	<0.0001	0.035	n.s.
CCL2	n.s.	0.022	n.s.	<0.0001
CCL5	<0.0001	<0.0001	0.001	<0.0001
CXCL10	0.038	0.002	0.003	0.001
CCR1	n.s.	<0.0001	0.003	0.003
CYBA	n.s.	<0.0001	n.s.	n.s.
CYBB	n.s.	<0.0001	0.044	n.s.
NRF2	n.s.	0.001	0.001	n.s.
GPX1	0.009	<0.0001	0.006	0.006
HMOX1	n.s.	<0.0001	n.s.	0.002
GBP1	n.s.	0.001	0.022	n.s.
GBP2	<0.0001	<0.0001	<0.0001	<0.0001
GBP4	0.019	0.016	0.003	n.s.
GBP5	0.014	0.001	0.017	0.006
TLR2	<0.0001	<0.0001	0.001	n.s.
TLR7	n.s.	<0.0001	<0.0001	n.s.

*Gene expression data were obtained in samples microdissected from 5 non-pathological control brains (5 WM and 6 GM areas) and 10 MS brains (14 NAWM, 13 actively demyelinating WM lesions, 8 chronic active WM lesions and 24 subpial chronic GM lesions). Figures represent p values for statistically significant differences in gene expression between control and MS brain parenchymal samples, as assessed by Mann-Whitney test (p < 0.05 without Bonferroni correction; when applying the Bonferroni correction, the significance threshold was p < 0.017); n.s., not significant*.

Genes that were significantly more expressed in the MS NAWM than in control WM include: the transcription factors IRF1 and IRF8 which play a key role in the amplification of the macrophage response to IFNγ (Langlais et al., 2016; Platanitis and Decker, 2018); IRF4, known to promote an anti-inflammatory macrophage phenotype (Gunthner and Anders, 2013); IFNγ and IFNβ inducible genes (IFI16 and MxA, respectively); genes involved in signaling to microglia (TREM2, CSF1R) (Colonna and Butovsky, 2017), antigen presentation (cathepsin S, RFX5, CIITA; HLA-DRA), T-cell costimulation (CD86), antigen presenting cell (APC) activation (CD40), and phagocytosis (CD68, MSR1); caspase 1, the enzyme involved in IL1 and IL18 secretion and pyroptosis, a potent innate immune effector mechanism used to clear intracellular pathogens (Man et al., 2017); the proinflammatory cytokines IL16 and IL18; TNFR1 and TNFR2 mediating pro-inflammatory and neuroprotective TNF responses, respectively (Brenner et al., 2015); the immunosuppressive cytokines IL10 and TGFβ1; the IFNγ-inducible chemokines CXCL10 and CCL5 promoting lymphocyte and myeloid cell recruitment; GPX1, a major antioxidant enzyme (Vilhardt et al., 2017); IFNγ-inducible GBPs, GBP2, GBP4, and GBP5, involved in anti-microbial responses and inflammasome activation (Kim et al., 2016); the pattern recognition receptor TLR2 (Table 3). These data indicate that signals delivered by CNS-infiltrating immune cells stimulate a complex innate immune response already in the NAWM, which manifests with increased expression of genes involved in classical microglia/macrophage anti-microbial functions and in homeostatic functions.

A greater number of genes were upregulated in the active WM lesion parenchyma surrounding a heavily infiltrated central vein and containing myelin-engulfing macrophages (Table 3). Except for IL18, all the genes upregulated in the NAWM were also found to be significantly more expressed in actively demyelinating WM lesions than in the control WM. Additionally, active WM lesions were characterized by increased expression of: RUNX1, a transcription factor activating macrophage differentiation genes (Butovsky and Weiner, 2018); IFNβ, STAT1, STAT2, IFI6, and OAS1 involved in IFN signaling (Platanitis and Decker, 2018); the B-cell growth factor BAFF, a cytokine produced by astrocytes (Krumbholz et al., 2005) and microglia (Kim et al., 2009); the monocyte chemoattractant CCL2; the CCL5 receptor CCR1; CYBA and CYBB, encoding p22phox and gp91phox/NOX2, respectively, two components of the superoxide-generating nicotinamide adenine dinucleotide phosphate (NADPH) complex (Vilhardt et al., 2017); NRF2 and HMOX1, counteracting production of reactive oxygen species (Vilhardt et al., 2017); the IFNγ-inducible GBP1 and TLR7. Collectively, the transcriptional profile of active WM lesions suggests amplification of IFN-regulated microglia/macrophage responses and induction of pro-oxidant activity in concomitance with accentuated anti-inflammatory and anti-oxidant responses.

Although being considerably less numerous (Table 3), the genes showing enhanced expression at the border of chronic active WM lesions overlapped with those induced in active WM lesions and included: TREM2; RUNX1; IRF4 associated with an anti-inflammatory macrophage phenotype; IRF8 and IFI16, involved in IFNγ pathway activation; caspase 1; genes involved in antigen presentation (cathepsin S, HLA-DRA), T-cell (CD86) and APC (CD40) stimulation, phagocytosis (CD68, MSR1), immunosuppression (TGFβ1), TNF-mediated neuroprotection (TNFR2), immune cell recruitment (CCR1, CCL5, CXCL10), pro-oxidant (CYBB) and antioxidant (NRF2, GPX1) activity, antimicrobial response (GBP2, GBP4, GBP5), and pathogen recognition (TLR2, TLR7). These results suggest reduced microglia/macrophage activation at the site of chronic WM lesion expansion; still, the pattern of induced genes is consistent with ongoing pro-inflammatory and counter-regulatory functional states.

We next evaluated possible differences in gene expression among the WM areas isolated from the MS brain. Using the Kruskal-Wallis test and *post-hoc* Dunn's test with Bonferroni correction (significance threshold *p* < 0.0125), we identified a few differentially expressed genes between the NAWM and active WM lesions. Both genes involved in the pro-inflammatory cascade [IL6 (*p* = 0.008), BAFF (*p* = 0.004), CCR1 (*p* = 0.011)] and genes encoding molecules associated with a macrophage healing response [IRF4 (*p* = 0.008), CD163 (*p* = 0.004), the antioxidant molecules NRF2 (*p* = 0.004), GPX1 (*p* = 0.008), HMOX1 (*p* = 0.011)] were significantly more expressed in active WM lesions than in the NAWM. No differentially expressed genes were identified between chronic active WM lesions and NAWM or active WM lesions.

Genes that were upregulated in chronic subpial GM lesions contiguous to prominent meningeal immune infiltrates in comparison to the control GM include the microglia homeostatic marker TMEM19 (Butovsky and Weiner, 2018); IFNβ; the IFNγ-inducible transcriptional activator IFI16; genes involved in antigen presentation (cathepsin S, CIITA, HLA-DRA), APC activation (CD40), phagocytosis (CD163, CD68), proinflammatory cytokine production (caspase 1, IL1β, IL18), B-cell growth (BAFF), TNF-mediated pro-inflammatory activity (TNFR1), lymphocyte and myeloid cell chemotaxis (CCR1, CCL2, CCL5, CXCL10), anti-oxidant (GPX1, HMOX1), and antimicrobial (GBP2, GBP5) activities (Table 3). Despite lack of induction of many genes involved in microglia/macrophage regulation and function is consistent with modest microglia activation in chronic subpial GM lesions, the transcriptional profile of this type of lesions suggests a distinctive microglia phenotype, characterized by increased expression of genes encoding pro-inflammatory cytokines and chemokines in the absence of significant induction of anti-inflammatory genes.

**Figure 3:**
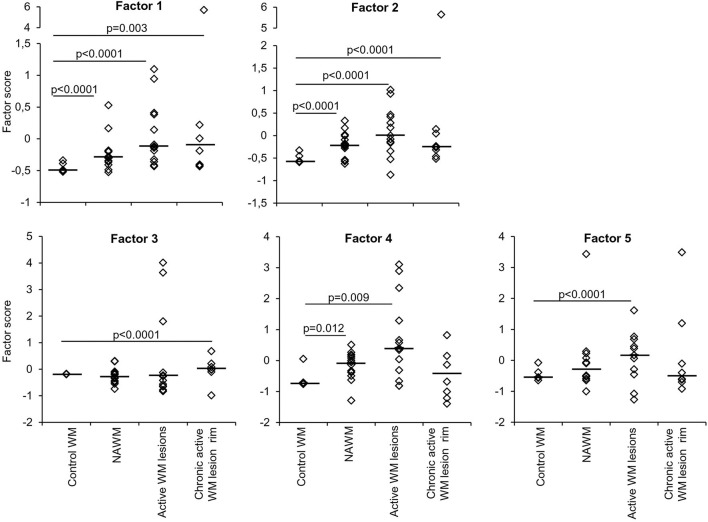
Discriminating power of artificial factors derived from gene expression data of microdissected white matter from control and MS brains. Differences in factor scores between control and MS WM parenchymal samples are shown. Factor 1 and Factor 2 scores strongly discriminate between control WM and all the MS WM areas analyzed [Factor 1: NAWM, AUC 0.84 (95% CI 0.66–1); active WM lesions, AUC 0.92 (95% CI 0.8–1); chronic active WM lesion rim AUC 0.85 (95% CI 0.62–1); Factor 2: NAWM, AUC 0.86 (95% CI 0.68–1); active WM lesions, AUC 0.88 (95% CI 0.71–1); chronic active WM lesion rim, AUC 0.9 (95% CI 0.72–1)]. Factor 3 well discriminates between control WM and chronic active WM lesion rim [AUC 0.88 (95% CI 0.63–1)]. Factor 4 discriminates between control WM and both NAWM [AUC 0.81 (95% CI 0.55–1)] and active WM lesions [AUC 0.8 (95% CI 0.58–1)]. Factor 5 well discriminates between control WM and active WM lesions [AUC 0.8 (95% CI 0.59–1)]. Each dot represents the mean factor score value for each WM sample analyzed; the line marks the median value. Statistically significant differences between groups were assessed by Mann-Whitney test (*p* < 0.05, without Bonferroni correction; *p* < 0.01 with Bonferroni correction).

Factor analysis on gene expression data obtained from control and MS WM samples identified five artificial (or empirical) factors that explained 28.2, 18.0, 11.2, 9.4, and 8.3% (in total 75.1%) of the variability in the dataset. Table 4 shows the genes with the strongest correlation (factor loadings >0.5) with each factor. Factor 1 comprised IRF1 and IRF4 and genes involved in IFN signaling, antigen presentation, phagocytosis, anti-microbial function, pro- and anti-inflammatory cytokine production and pro- and anti-oxidant activities, highlighting the coordinated expression of genes related to classical macrophage activation and healing responses. Factor 2 comprised genes involved in IFN signaling, antigen presentation and phagocytosis, as well as caspase 1, TNFR2, CCR1, GPX1, and TLR7. Scores for Factor 1 and Factor 2 were higher in all MS WM areas analyzed than in control WM, and both factors had a high discriminating power in all comparisons (AUC > 0.80 by ROC curve analysis) (Figure 3). Factor 3 comprised IFNβ, microglia/macrophage chemokine receptors (CCR1, CCR2, CX3CR1) and TLR2 and discriminated well the chronic active WM lesion rim from control WM. Factor 4 comprised IRF8, CD86, CXCL16, and CYBA and discriminated well NAWM and active WM lesions from control WM, while Factor 5 comprised STAT1, CCL5, and GBP1 and discriminated well active WM lesions from control WM (Figure 3), highlighting the link between IFNγ pathway activation and IFNγ-inducible genes mediating pro-inflammatory and anti-microbial microglia/macrophage responses. None of the five factors accurately discriminated between NAWM, active WM lesions and chronic active WM lesion rim, supporting absence of major differences in the transcriptional programs activated in the different MS WM areas.

**Table 4:** Factor loadings derived from gene expression data of control and MS WM parenchyma.

**Gene**	**Factor 1**	**Factor 2**	**Factor 3**	**Factor 4**	**Factor 5**
CSF1R					
RUNX1	0.92				
IRF1	0.81				
IRF4	0.92				
IRF8				0.7	
IFNβ			0.92		
JAK2		0.91			
STAT1					0.8
STAT2	0.52	0.66			
IFI16	0.68	0.54			
MXA					
OAS1		0.84			
Cathepsin S	0.56	0.56			
HLA-DRA		0.6			
CIITA	0.88				
CD86				0.87	
CD68	0.59	0.68			
MSR1	0.76				
CD163	0.76				
CXCL16	0.63			0.52	
NLRP3					
Caspase 1	0.53	0.77			
GBP1					0.89
GBP2	0.81				
GBP5					
TNFR1					
TNFR2		0.62			
IL1β	0.93				
TGFβ1	0.76				
CCL5					0.87
CCR1		0.63	0.64		
CCR2			0.93		
CX3CR1			0.61		
CYBB	0.89				
CYBA				0.57	
NRF2	0.63				
GPX1	0.64	0.55			
HMOX1	0.78				
TLR2			0.93		
TLR7		0.82			

*Factor loadings > 0.5 in absolute value are shown*.

Factor analysis on data obtained in laser-cut GM samples yielded artificial factors with no discriminating power between control GM and chronic subpial GM lesions (data not shown).

### TNFR2 Protein Expression in the MS Brain White Matter and Gray Matter

**Figure 4:**
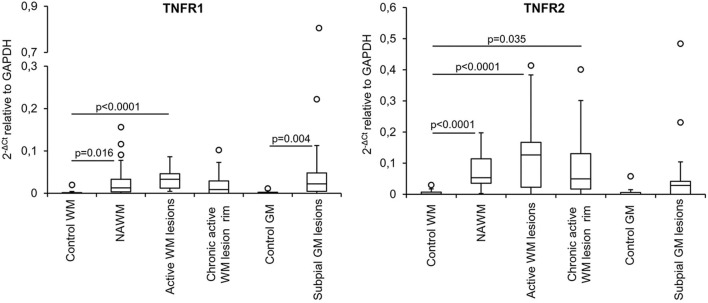
TNFR1 and TNFR2 gene expression in microdissected white matter and gray matter from control and MS brains. The graphs depict the differences in TNFR1 and TNFR2 gene expression between the indicated control and MS brain parenchymal areas. Data are expressed as 2^−ΔCt^ relative to the housekeeping gene GAPDH. Comparisons between control and MS WM and GM areas were performed using the Mann-Whitney test; statistically significant differences (*p* < 0.017 after Bonferroni correction for WM comparisons and *p* < 0.05 for GM comparison) are shown. The lines inside the boxes represent the median value; boxes extend from the 25th to the 75th percentile, covering the interquartile range (IQR), and whiskers extend from the 25th percentile−1.5 IQR to the 75th percentile + 1.5 IQR. Maximum outliers outside the whiskers are represented by individual marks.

The above data highlighted NAWM and lesion-specific induction of TNFR1 and TNFR2 gene expression in the vicinity of CNS immune infiltrates (Table 3, Figure 4). Furthermore, only TNFR2, but not TNFR1, correlated strongly with genes involved in macrophage effector functions (Factor 2) (Table 4). Because increasing evidence supports a neuroprotective role for TNFR2-mediated TNF signaling in different experimental models of CNS injury (Suvannavejh et al., 2000; Arnett et al., 2001; Fontaine et al., 2002; Brambilla et al., 2011; Taoufik et al., 2011; Patel et al., 2012; Probert, 2015; Dong et al., 2016; Madsen et al., 2016; Gao et al., 2017), we deemed important to define precisely the cellular localization of TNFR2 and its relation to lesion evolution in the MS brain using immunohistochemical techniques. TNFR2 immunoreactivity was examined in the subcortical WM and in the cortical GM of four cases without neurological disease and 11 cases with progressive MS, five of which were also included in the gene expression study (Table 1).

**Figure 5:**
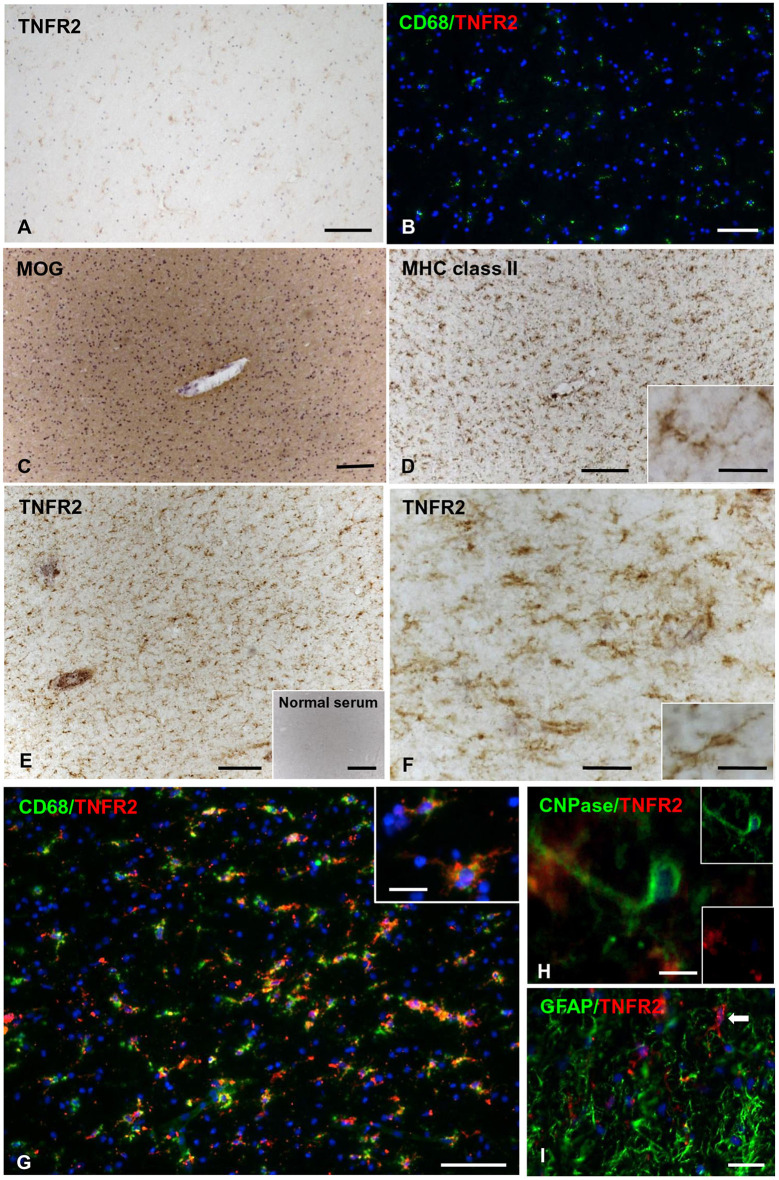
TNFR2 protein expression in the normal-appearing white matter in the MS brain. Immunostaining for TNFR2 **(A)** and double immunofluorescence for CD68 (green) and TNFR2 (red) **(B)** reveal absence of TNFR2 immunoreactivity in the non-pathological subcortical WM of two control cases (C25 and C30, respectively). Immunostainings for MOG **(C)** and MHC class II antigen **(D)** show intact myelin and widespread microglial activation in the NAWM surrounding an inflamed blood vessel (MS100 case); the inset in **(D)** shows a ramified MHC class II+ microglial cell at high magnification. Staining of an adjacent section for TNFR2 **(E,F)** shows immunoreactivity in numerous cells with microglial morphology throughout the same area. Absence of staining after incubation of an adjacent brain section with normal serum is shown in the inset in **(E)**. The inset in **(F)** shows a TNFR2+ cell with a ramified morphology at high magnification. Double immunofluorescence staining for CD68 (green) and TNFR2 (red) reveals presence of many CD68+ microglial cells co-expressing TNFR2 in the NAWM **(G)**. In the inset in **(G)**, two CD68+ TNFR2+ microglial cells are shown at high magnification. Double immunofluorescence stainings for TNFR2 and CNPase **(H)** or GFAP **(I)** reveal absence of TNFR2 immunoreactivity in oligodendrocytes and astrocytes in the NAWM. Sections in **(A,C–F)** are counterstained with hematoxylin. Nuclei are stained with DAPI (blue) in **(B,G–I)**. Bars: 500 μm in the inset in **(E)**; 200 μm in **(A,C,D)**; 100 μm in **(B,E,G)**; 50 μm in **(F)**; 20 μm in **(I)** and insets in **(D,F,G)**; 10 μm in **(H)**.

**Figure 6:**
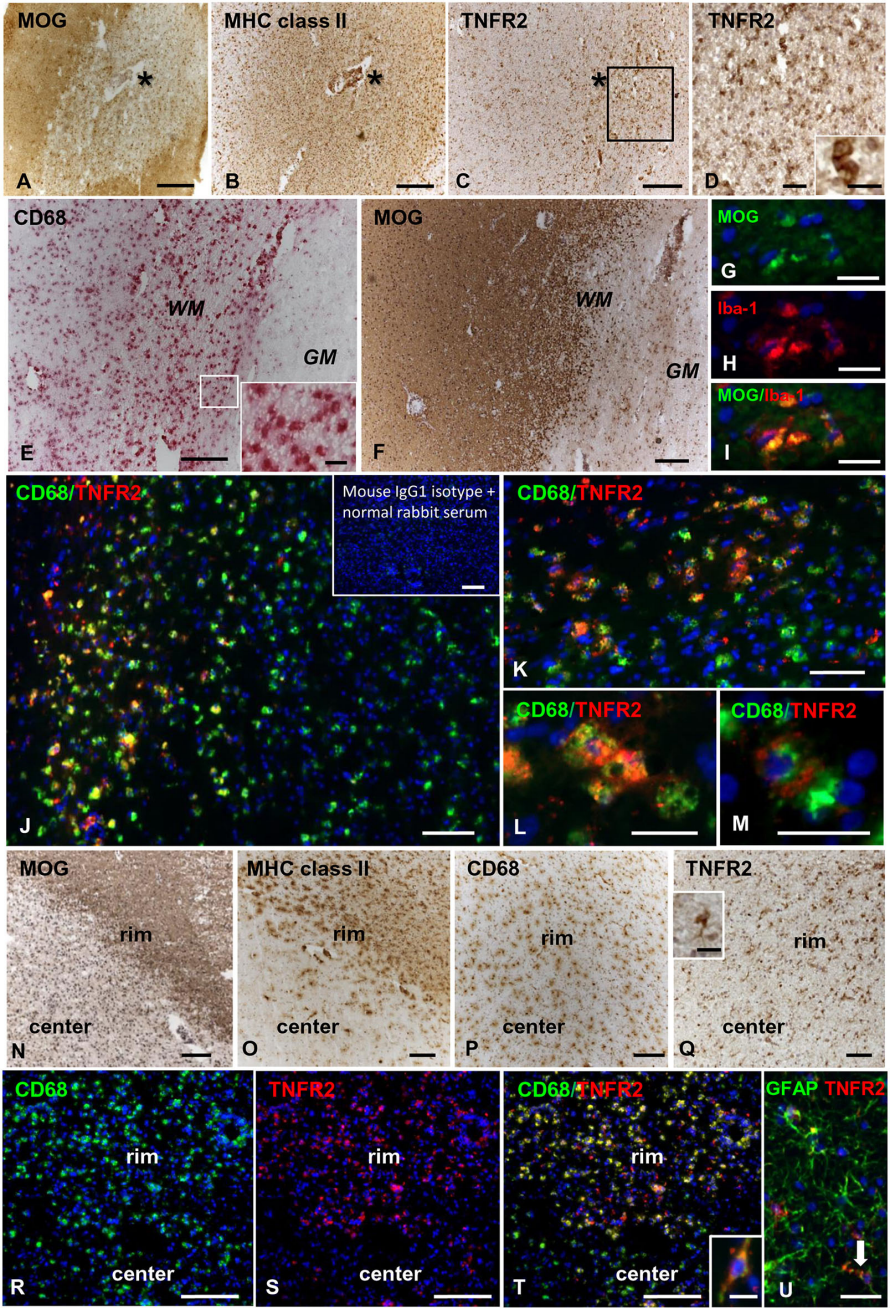
TNFR2 protein expression in actively demyelinating and chronic active white matter lesions in the MS brain. Immunostainings for MOG **(A)**, MHC class II antigen **(B)**, and TNFR2 **(C)** in serial MS brain sections (MS180 case) show an actively demyelinating WM lesion containing numerous TNFR2+ cells with a macrophage-like morphology; the asterisk marks the perivascular immune infiltrate in the center of the lesion (barely visible in the section stained for TNFR2). The frame in **(C)** marks the area shown at higher magnification in **(D)**; the inset in **(D)** shows two macrophage-like TNFR2+ cells. Immunostainings for CD68 **(E)** and MOG **(F)** in serial brain sections of case MS121 show the presence of foamy macrophages throughout a subcortical actively demyelinating WM lesion; the frame in **(E)** marks the area shown at high magnification in the inset. Double immunofluorescence staining for MOG (green, **G**) and the macrophage marker Iba-1 (red, **H**) shows Iba-1+ macrophages containing MOG (merge, **I**). Double immunofluorescence staining for CD68 (green) and TNFR2 (red) **(J,K)** reveals the presence of numerous CD68+ macrophages expressing TNFR2 in the same area shown in **(E,F)**; TNFR2+ CD68+ foamy macrophages are shown at high power magnification in **(L,M)**. Inset in **(J)** shows absence of staining in an adjacent brain section after replacement of primary antibodies with a mixture of preimmune rabbit serum and unconjugated mouse IgG1. MOG **(N)**, MHC class II antigen **(O)**, and CD68 **(P)** immunostainings in brain sections of MS79 case identify a subcortical chronic WM lesion with an active border (rim) and an inactive, demyelinated core (center). CD68 immunoreactivity is present throughout the lesion whereas MHC class II+ cells localize in the lesion border. Immunostaining for TNFR2 **(Q)** reveals that this receptor is expressed in many cells with microglia and macrophage morphologies in the lesion border; the inset in **(Q)** shows one TNFR2+ cell displaying the typical microglial morphology at high magnification. Double immunofluorescence staining for CD68 (green, **R**) and TNFR2 (red, **S**) in a serial section confirms the expression of TNFR2 in numerous CD68+ microglial cells and macrophages in the active border of the chronic WM lesion (merge, **T**); one CD68+ TNFR2+ cell with a bipolar morphology is shown at high magnification in the inset in **(T)**. Double immunofluorescence for GFAP (green) and TNFR2 (red) **(U)** reveals the presence of an occasional TNFR2+ astrocyte (arrow) in the rim of the same chronic active WM lesion. Sections in **(A–F)** and **(N–Q)** are counterstained with hematoxylin; nuclei are stained with DAPI (blue) in **(G–M)** and **(R–U)**. Bars: 200 μm in **(A–C,E,F,N–P)** and inset in **(J)**; 100 μm in **(Q,R–T)**; 50 μm in **(D,J)**; 20 μm in **(G–I,K–M,U)** and insets in **(D,Q)**; 10 μm in the inset in **(T)**.

In control brains, no TNFR2 immunoreactivity was observed in the WM and GM without evidence of microglia activation (Figures 5A,B). In MS brain samples, numerous TNFR2+ cells with a ramified morphology were observed in areas of the NAWM characterized by massive microglia activation, as assessed by MHC class II immunostaining, both close to and far from perivascular immune cell infiltrates (Figures 5C–F). Double immunofluorescence staining with anti-TNFR2 and anti-CD68 confirmed that nearly all TNFR2+ cells (tentatively 93–97%) co-expressed CD68 (Figures 5G–I); the percentage of CD68+ TNFR2+ cells in the CD68+ cell population was more variable, ranging from 25 to 75% (median value 39%) in different MS brain samples (*n* = 5). TNFR2 immunoreactivity was also detected in many cells throughout active WM lesions (*n* = 4) (Figures 6A–D). Double immunofluorescence staining showed that TNFR2 immunoreactivity was restricted to CD68+ foamy macrophages (Figures 6E–M); the percentage of CD68+ TNFR2+ cells ranged between 24 and 39% (median value 26%) of the intralesional CD68+ cell population. TNFR2+ CD68+ cells with the morphology of activated microglia and macrophages were also detected at the edge of chronic active WM lesions (*n* = 7) (Figures 6N–T), representing 75–88% (median value 84%) of TNFR2+ cells and 17–89% (median value 36%) of CD68+ cells in different brain samples. The staining intensity and the number of TNFR2+ microglia-like cells markedly decreased from the rim toward the MHC class II-negative, demyelinated center of the lesion (Figures 6N–Q). In the inactive lesion center, TNFR2+ CD68+ cells represented 60–73% (median value 63%) of TNFR2+ cells and 1.4–20% (median value 5.6%) of CD68+ cells. TNFR2 immunoreactivity was not detected in CNPase+ oligodendrocytes and GFAP+ astrocytes in the NAWM (Figures 5H,I, respectively) and in active WM lesions (not shown). Some TNFR2+ GFAP+ astrocytes were only observed at the border of chronic active WM lesions, representing 1 to 7% (median value 3%) of total GFAP+ cells (Figure 6U).

**Figure 7:**
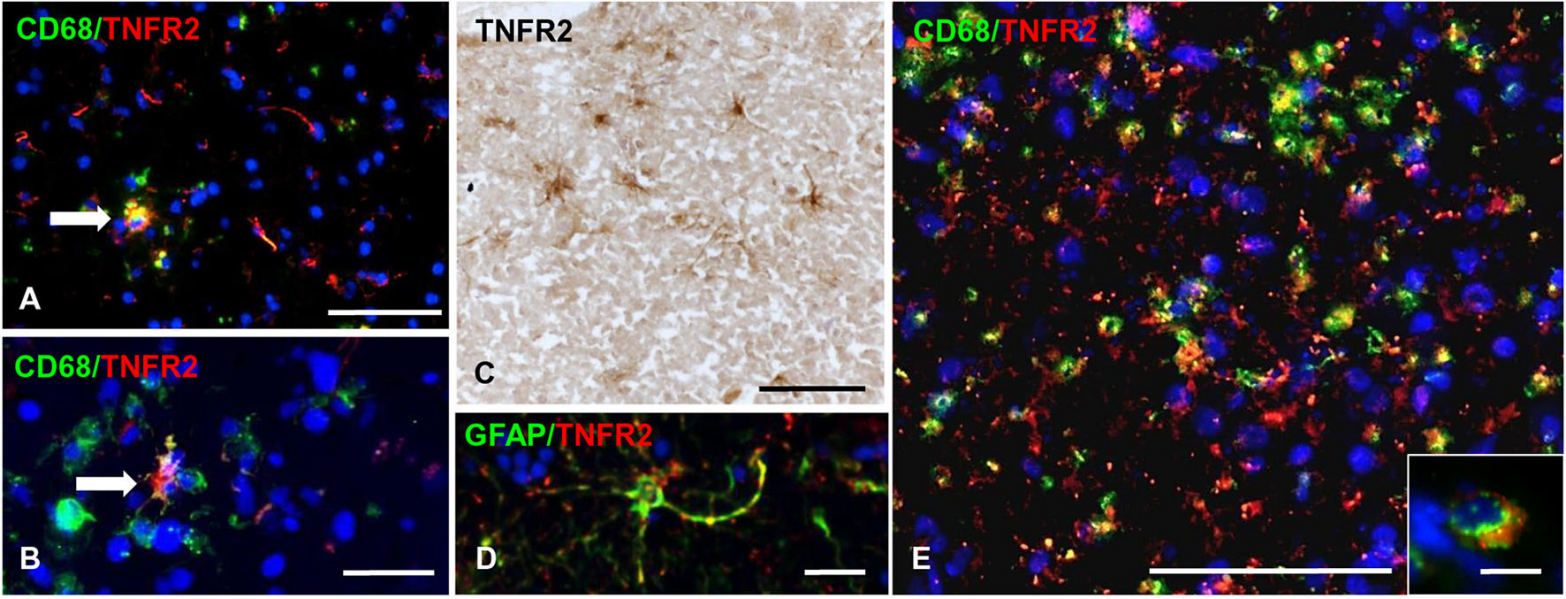
TNFR2 protein expression in subpial gray matter lesions. Rare TNFR2+ CD68+ cells with macrophage (**A**, arrow) or microglia morphology (**B**, arrow) are present at the edge of two different chronic active subpial GM lesions (MS160 and MS180 case, respectively). TNFR2 immunoreactivity is observed in cells resembling reactive astrocytes in a chronic active subpial GM lesion (MS79 case) **(C)**; a TNFR2+ GFAP+ astrocyte present in the same area is shown in **(D)**. Double immunofluorescence staining for CD68 (green) and TNFR2 (red) shows presence of many CD68+ TNFR2+ cells with a macrophage morphology in an active subpial GM lesion (MS79 case) **(E)**; a TNFR2+ CD68+ macrophage is shown at high magnification in the inset. Bars: 50 μm in **(A,C,E)**; 20 μm in **(B,D)**; 10 μm in the inset in **(E)**.

TNFR2 immunoreactivity was almost absent in the NAGM and in chronic inactive subpial GM lesions where microglia was largely CD68-negative (data not shown). At the border of chronic active subpial GM lesions (*n* = 7), a few TNFR2+ cells were detected and identified as CD68+ foamy macrophages and ramified microglia (Figures 7A,B). Twenty-four to 50% (median value 42.8%) of CD68+ cells co-expressed TNFR2. TNFR2+ GFAP+ astrocytes were also present at the border of some chronic active subpial GM lesions (Figures 7C,D) in four out of seven MS cases examined. Quantification of TNFR2+ GFAP+ was however difficult; possibly, <15% of GFAP+ astrocytes co-expressed TNFR2 in the lesion rim. Only within two active and demyelinating subpial GM lesions of MS160 case, a very rare finding in chronic MS, a high density of TNFR2+ CD68+ foamy macrophages and activated microglia was observed (Figure 7E); TNFR2+ CD68+ cells accounted for 29 to 50% (median value 43%) of intralesional CD68+ cells.

### TNFR2 Expression and Functionality During Remyelination in an *ex vivo* Demyelinating Model

**Figure 8:**
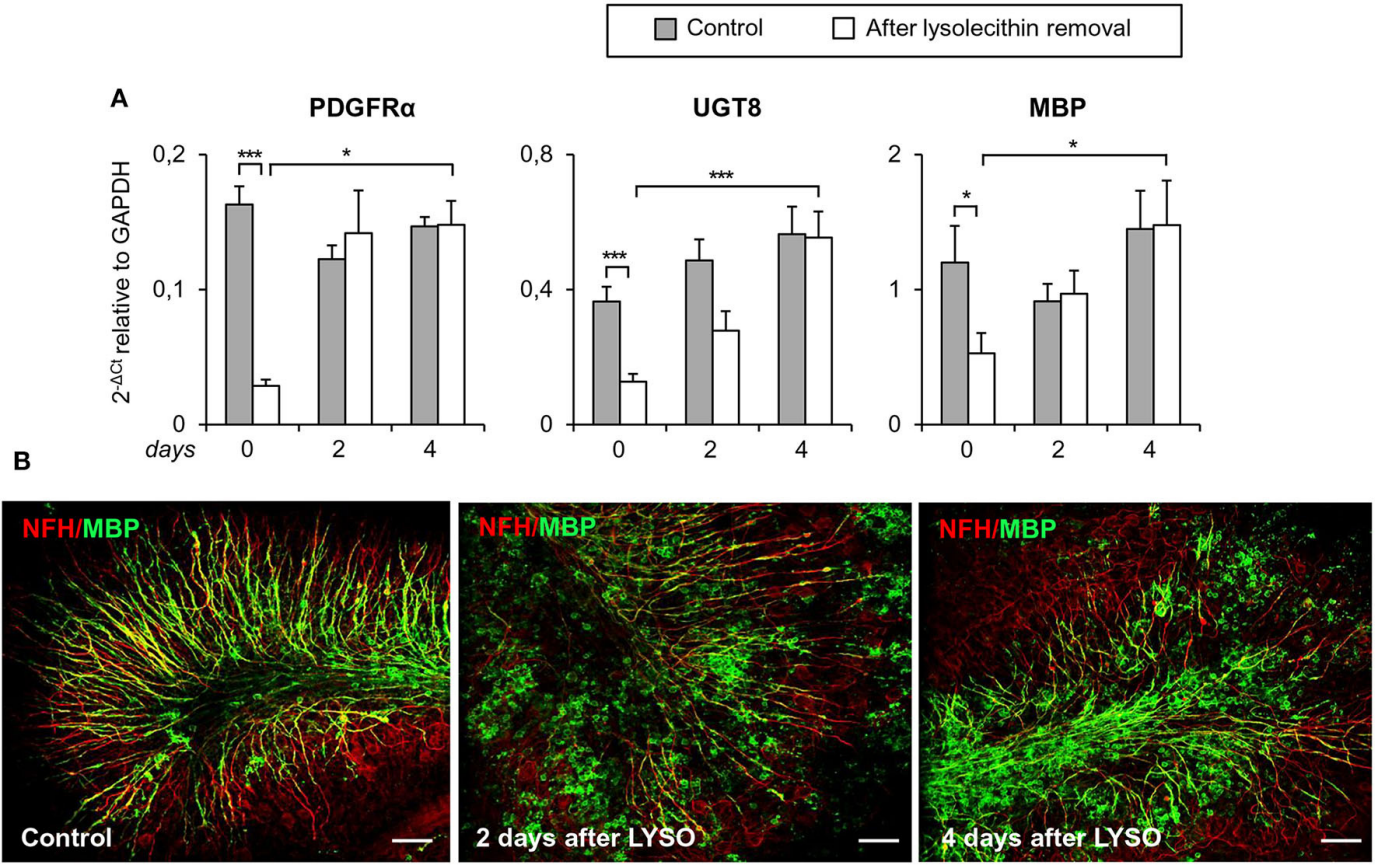
Demyelination and remyelination in mouse cerebellar slices. Mouse cerebellar slices maintained *in vitro* for 7 days were grown in the absence or presence of lysolecithin (0.5 mg/ml) for 16 h and then cultured in normal medium for the indicated time. **(A)** Cerebellar slices were collected immediately (0 days), and at 2 days and 4 days after toxin removal. RNA was extracted and reverse transcribed and the expression of the indicated oligodendrocyte differentiation-related genes was investigated using real time RT-PCR. Data are expressed as 2^−ΔCt^ relative to GAPDH. Means ± SEM of five experiments are shown. ^*^p < 0.05; ^***^p < 0.001 by 2-way ANOVA with *post-hoc* Bonferroni correction. **(B)** Double immunostaining for MBP (green) and NFH (red). After 7 days in culture, a considerable myelin and axon alignment is evident in control slices. At 2 days after lysolecithin removal, loss of MBP is observed; after 4 days of recovery, MBP immunoreactivity is increased and its alignment with axons is evident. One representative experiment out of three performed is shown. Bars = 50 μM.

The timing of TNFR2 expression in response to myelin damage was studied in organotypic mouse cerebellar slices following lysolecithin-induced demyelination. This experimental model has the advantage of preserving the CNS cytoarchitecture while showing spontaneous remyelination and microglia activation after toxin removal (Barateiro et al., 2016). Cerebellar slices from P10 mice were cultured for 7 days and then treated for 16 h with lysolecithin to induce demyelination, as previously described (Eleuteri et al., 2017). In this model, the level of transcripts associated with oligodendrocyte lineage development (PDGFRα, UGT8, MBP) decreases immediately after treatment with lysolecithin (day 0) and rapidly increases during the following 4 days (Figure 8A). During this period, some degree of remyelination occurs as shown by double immunostaining with anti-MBP and anti-NFH antibodies (Figure 8B).

**Figure 9:**
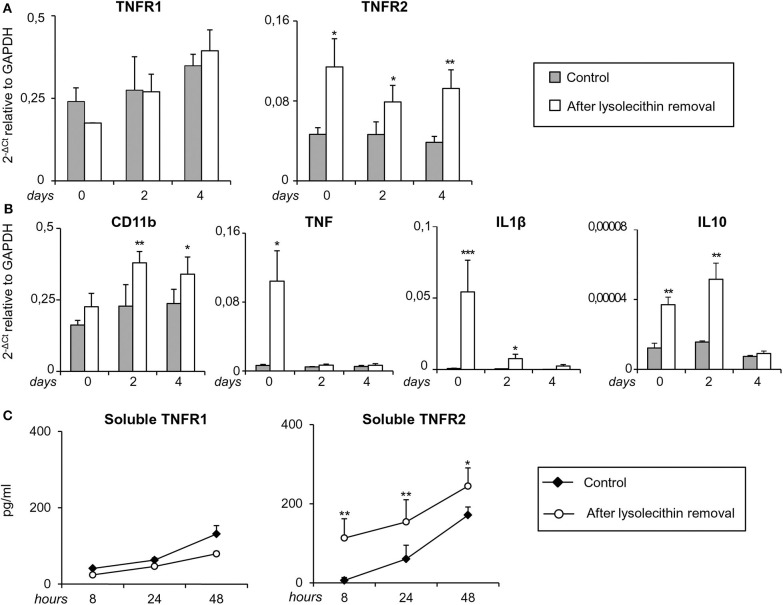
Regulation of TNF receptors and microglia/macrophage markers during demyelination and remyelination in mouse cerebellar slices. Mouse cerebellar slices maintained *in vitro* for 7 days were grown in the absence or presence of lysolecithin (0.5 mg/ml) for 16 h and then cultured in normal medium. **(A,B)** Slices were collected immediately (0 days), at 2 days and 4 days after toxin removal. RNA was extracted and reverse transcribed and the expression of the indicated genes was investigated using real time RT-PCR. Data are expressed as 2^−ΔCt^ relative to GAPDH. **(C)** Culture supernatants were collected at 8, 24, and 48 h after toxin removal and the amount of soluble TNFR1 and TNFR2 was measured using specific ELISA. Means ± SEM of 3 experiments are shown. ^*^p < 0.05; ^**^p < 0.01; ^***^p < 0.001 by Student's *t*-test.

At the same time points, TNFR1 and TNFR2 transcripts were analyzed together with transcripts for the microglia/macrophage activation marker CD11b, the pro-inflammatory cytokines IL1β and TNF, and the anti-inflammatory cytokine IL10. As shown in Figure 9A, TNFR2, but not TNFR1, RNA levels were significantly higher in toxin-treated cerebellar slices than in untreated slices at all-time points examined (0, 2, and 4 days after toxin removal). As to the microglia/macrophage response elicited by lysolecithin, CD11b was significantly and stably induced at 2 and 4 days after toxin removal. Concomitantly, both pro- and anti-inflammatory cytokines were up-regulated but with different kinetics (Figure 9B). Transcripts for TNF and IL1β peaked at the time of toxin removal and were no longer and barely detectable, respectively, after 2 days, namely during the remyelination phase. IL10 RNA was also rapidly induced but its level remained stable until 2 days after toxin removal (Figure 9B).

Since release of soluble TNFR2 is an important component of the anti-inflammatory response of macrophages (Jin et al., 2000), regulatory T cells (van Mierlo et al., 2008) and microglia (Veroni et al., 2010), shedding of both TNFRs was investigated after myelin damage using specific ELISA. The amount of soluble TNFR1 in the culture medium did not differ between control and lysolecithin-treated cerebellar slices at any time point examined (Figure 9C). Conversely, the amount of soluble TNFR2 significantly increased between 8 and 48 h after toxin removal (Figure 9C). These results confirmed absence of TNFR1 regulation and sustained induction of TNFR2 that accompanies the shift from a mixed pro-/anti-inflammatory microglia/macrophage phenotype to a predominantly anti-inflammatory phenotype during the recovery phase.

**Figure 10:**
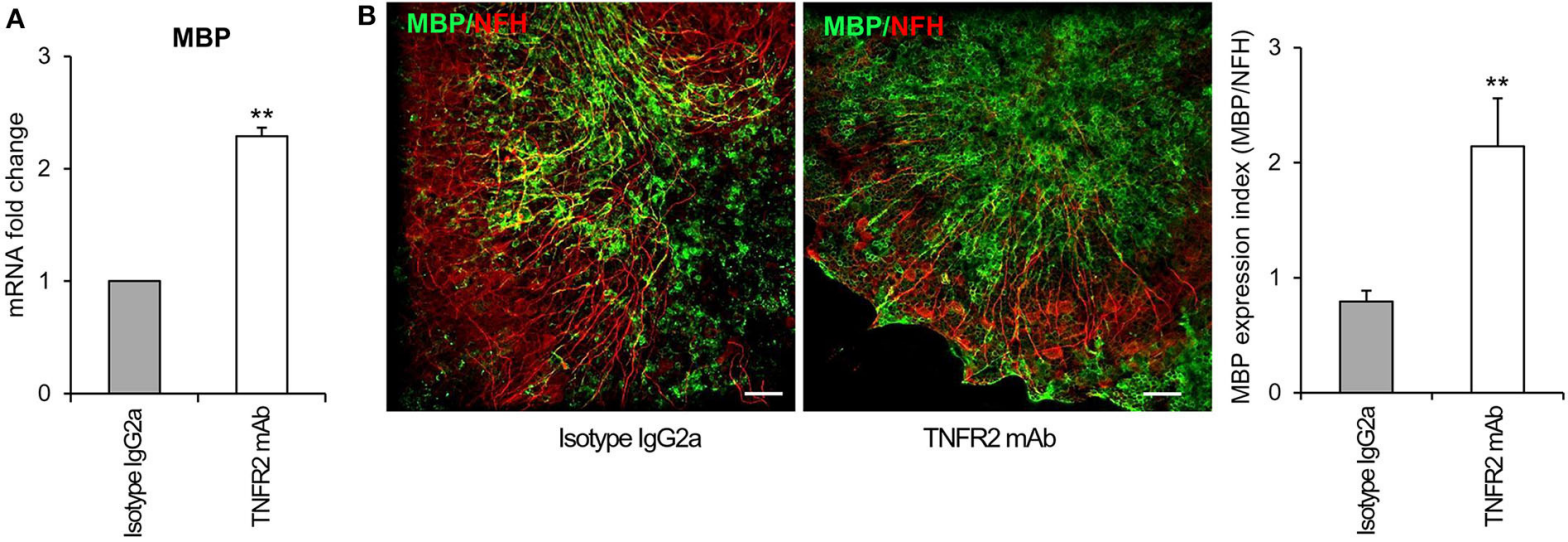
Effect of selective TNFR2 activation on MBP expression in remyelinating cerebellar slices. Cerebellar slices from P10 mice were grown for 7 days *in vitro* and then treated with lysolecithin (0.5 mg/ml) for 16 h. Medium was then replaced with fresh medium containing TNFR2 mAb (2 μg/ml) or rat IgG2a (2 μg/ml) as isotype control. **(A)** After 4 days slices were collected, RNA was extracted and reverse transcribed and MBP RNA expression was assessed by real time RT-PCR. Data are expressed as fold increase of each RNA (normalized to GAPDH) in TNFR2 mAb-treated slices relatively to isotype control slices (2^−ΔΔCt^). **(B)** After 7 days *in vitro* cerebellar slices were immunostained for MBP and NFH and the index of MBP protein expression was calculated as the ratio between the immunofluorescence intensity of MBP and NFH; one representative experiment of 3 performed is shown. Means ± SEM of three independent experiments are shown; ^**^p < 0.01 by Student's *t*-test.

To investigate the functionality of TNFR2 in the cerebellar slice model, we verified whether selective TNFR2 stimulation could promote the spontaneous regenerative process after myelin damage. Slices were exposed to lysolecithin and then incubated in the presence of an agonistic TNFR2-specific mAb (2 μg/ml) that was previously shown to induce neuroprotection in primary mouse cortical neurons (Marchetti et al., 2004) and to increase expression of genes encoding anti-inflammatory and neuroprotective cytokines in cultured mouse microglia (Veroni et al., 2010). Rat IgG2a was used as isotype control. After 4 and 7 days of treatment, slices were examined for MBP gene and protein expression, respectively. TNFR2 mAb caused a significant increase both in MBP RNA level (Figure 10A) and in the index of MBP expression (quantified as the ratio between MBP and NFH immunofluorescence intensity) (Figure 10B), confirming the stimulating activity of TNFR2 signaling on oligodendrocyte maturation (Arnett et al., 2001).

## Discussion

Advanced analyses of MS genetic data implicate different peripheral immune cell types and microglia as contributing to the earliest events that trigger MS (International Multiple Sclerosis Genetics Consortium, 2019). To understand more on the cross-talk between the immune intruders and resident microglia and on the contribution of the latter to CNS tissue destruction and healing processes, in this study we assessed changes in the expression of genes related to inflammation and microglia/macrophage functions in the neural parenchyma immediately adjacent to immune cell infiltrates in the MS brain.

To date, several studies have investigated the transcriptional profile of manually or laser-cut microdissected NAWM (Koning et al., 2007; Zeis et al., 2008; Hendrickx et al., 2017; Zrzavy et al., 2017; Elkjaer et al., 2019), WM lesions (Hendrickx et al., 2017; Zrzavy et al., 2017; Elkjaer et al., 2019), NAGM and GM lesions (Fischer et al., 2013; Magliozzi et al., 2019) from the brain of MS patients, using mainly unbiased approaches. None of the published studies could exclude the contribution of CNS-infiltrating immune cells to the reported RNA profiles. Indeed, in some studies the most upregulated genes in the MS brain samples were unequivocally lymphocyte-specific [for example, IFNγ (Koning et al., 2007) and immunoglobulins (Elkjaer et al., 2019; Magliozzi et al., 2019)]. While focusing on a restricted panel of genes involved in inflammation and microglia/macrophage functions, the present study has allowed to generate novel, clear-cut data on the CNS endogenous innate immune response to signals originating from blood-derived immune cells that accumulate and become activated inside the CNS.

A major finding of this study is that the transcriptional profile of the NAWM surrounding perivascular immune infiltrates is characterized by the enhanced expression of genes involved in a classical antimicrobial macrophage response and in macrophage-mediated tissue healing. Induction of genes involved in antigen presentation, T-cell stimulation, phagocytosis, pathogen recognition, antimicrobial activity, and leukocyte migration indicates that already in the early stages of microglia activation, and before active demyelination occurs, a typical macrophage defense program is induced. Such a complex program aims at stimulating recruitment and local activation of adaptive immune cells, at directly eliminating invading pathogens or infected cells, and at getting rid of dead cells and cell debris to promote tissue remodeling. Notably, several genes induced in the NAWM mediate IFNγ signaling (IRF1, IRF8) or are IFNγ-regulated genes (cathepsin S, RFX5, CIITA, HLA-DRA, IFI16, CD40, CD86, CCL5, CXCL10, GBP2, GBP4, GBP5) (Langlais et al., 2016), suggesting that IFNγ released by CNS-infiltrating, locally reactivated T cells plays a key role in driving early microglia activation. On the other hand, the NAWM close to perivascular cuffs is also characterized by upregulation of genes that are known to be associated with a protective microglia/macrophage phenotype, including TREM2 (Cantoni et al., 2015), CSF1R (Verreck et al., 2004), MSR1 (Martinez et al., 2006), IRF4 (Gunthner and Anders, 2013), TNFR2 (Veroni et al., 2010; Gao et al., 2017), IL10, TGFβ1, and GPX1 (Griess et al., 2020). These findings suggest that the extent of local T-cell activation can determine whether inflammatory or counter-regulatory microglial responses prevail in the non-demyelinated NAWM and hence the evolution into highly destructive demyelinating lesions.

While confirming increased expression of genes involved in antigen presentation (HLA-DRA), phagocytosis (CD68, MSR1) and immunoregulation (IL10) in the NAWM, this study differs from previous studies showing more limited or no changes in the expression of inflammation- and microglia-related genes in NAWM samples isolated from the brain of acute (Zrzavy et al., 2017), relapsing-remitting (Hendrickx et al., 2017) and progressive (Koning et al., 2007; Zeis et al., 2008; Elkjaer et al., 2019) MS cases, and in microglia isolated from the NAWM of progressive MS cases (van der Poel et al., 2019). A logical explanation for these discrepancies is the choice to analyze only the NAWM surrounding inflamed blood vessels and containing activated microglia, thus improving the detection of inflammation-associated genes.

Besides the genes upregulated in the NAWM, more genes linked to IFN pathway activation (IFNβ, STAT1, STAT2, IFI6, OAS1) and microglia/macrophage effector functions, in particular genes involved in lymphocyte activation (BAFF), macrophage recruitment (CCL2), CCL5 chemoattractant activity (CCR1), oxidative injury (CYBA, CYBB), and anti-microbial activity (GBP1) were induced in actively demyelinating WM lesions. The increased expression of genes encoding components of the superoxide-generating NADPH complex is in line with previous studies showing that inflammation-associated oxidative burst in activated microglia and macrophages is implicated in demyelination and free radical-mediated tissue injury in active WM lesions (Fischer et al., 2012; Schuh et al., 2014; Zrzavy et al., 2017). As shown previously (Zrzavy et al., 2017), iNOS RNA was not induced in any of the WM areas analyzed.

IFNγ has a key role in the activation of macrophage oxidative metabolism and antimicrobial activity (Nathan et al., 1983) and is a potent inducer of NAPDH oxidases, including CYBA and CYBB (Casbon et al., 2012; Hodny et al., 2015). IFNγ is mainly produced by Th1 cells, CD8 T cells and NK cells, all of which play a key role in the host response against tumors and intracellular pathogens. Because CD8+ T cells predominate, expand and have an activated cytotoxic phenotype in the MS brain (Babbe et al., 2000; Serafini et al., 2007; van Nierop et al., 2017; Machado-Santos et al., 2018), it is likely that this immune cell subset represents the major local source of IFNγ. Excessive IFNγ production can lead to uncontrolled superoxide generation by microglia/macrophages and this could be the major driving force for demyelination and neurodegeneration (Fischer et al., 2012; Schuh et al., 2014). Activated CD8 T cells produce several other soluble mediators that can cause direct injury to oligodendrocytes and neurons or prevent remyelination, including TNF (Agresti et al., 1996; Denic et al., 2013; Magliozzi et al., 2019) and lytic enzymes, in particular granzyme B (Haile et al., 2011). A local, deleterious “cytokine storm” and ongoing cytotoxic activity are both compatible with the concept that CD8+ T cells recruited to the MS brain might be activated by a persistent EBV infection in the CNS, more specifically by EBV-infected B cells accumulating in the CNS connectival spaces (Serafini et al., 2007, 2019; Veroni et al., 2018). Accordingly, EBV elicits very strong CD8 T cell responses that cause significant collateral tissue damage in EBV-associated immunopathologic diseases (Taylor et al., 2015). We propose that inappropriate activation of an anti-microbial defense program in microglia by IFNγ-producing CD8 T cells that try to get rid of EBV plays a major role in the initiation and amplification of bystander CNS tissue damage in MS.

Our study also shows that, despite ongoing demyelination and the shift toward a more inflammatory and pro-oxidant phenotype, genes encoding molecules associated with an anti-inflammatory microglia/macrophage phenotype (IRF4, TREM2, MSR1, TNFR2, TGFβ1, IL10) and with protection from oxidative stress (NRF2, GPX1, HMOX1) were concomitantly upregulated in active WM lesions compared to the control WM. Some counter-regulatory molecules (IRF4, CD163, NRF2, GPX1, HMOX1) were also found to be more expressed in active WM lesions than in the NAWM. In agreement with previous immunohistochemical studies showing expression of anti-inflammatory cytokines in CD163+ myelin-engulfing macrophages in MS lesions (Boven et al., 2006; Zhang et al., 2011), these findings suggest that counter-regulatory responses are active during the most destructive phase of CNS inflammation to contain the inflammatory process and limit lesion expansion.

The transcriptional changes detected at the edge of chronic active WM lesions reveal some reduction in IFN-mediated signaling and microglia/macrophage activation during chronic lesion expansion. Infact, at variance with what observed in active WM lesions, several genes linked to IFN pathway activation (IFNβ, IRF1, STAT1, STAT2, IFI6, MxA, OAS1) and to microglia/macrophage pro-inflammatory (IL16, BAFF, CCL2)/pro-oxidant (CYBA) and anti-inflammatory (CSF1R, CD163, IL10)/anti-oxidant (HMOX1) functions, were not significantly induced in the chronic active WM lesion rim compared to control WM. However, no significant differences in gene expression were found between active and chronic active WM lesions. The fact that genes related to both pro-inflammatory and counter-regulatory microglia/macrophage functions were still upregulated in chronic active WM lesions suggests that their balance may be crucial in determining whether chronic WM lesions expand or evolve into inactive lesions.

The presence of abundant inflammatory infiltrates and ectopic B-cell follicle-like structures in the subarachnoid space of patients with progressive MS has been associated with more severe GM damage, particularly in the subpial cortex, and with an earlier disease onset and worst clinical course (Magliozzi et al., 2007, 2010; Howell et al., 2011). Together with extensive subpial demyelination, the findings of glia limitans damage and of a gradient of neuronal loss and microglia activation from the pial membrane in the cerebral cortex of MS patients with prominent meningeal inflammation (Magliozzi et al., 2010) are compatible with diffusion of large amounts of pro-inflammatory cytokines and cytolytic enzymes produced by CD8+ T cells accumulating in the subarachnoid space and being continuously activated by a locally dysregulated EBV infection (Serafini et al., 2007, 2019; Veroni et al., 2018). Our study shows that the transcriptional profile of chronic subpial GM lesions isolated from MS brain samples with prominent meningeal inflammation is consistent with skewing toward a detrimental, proinflammatory environment and microglia phenotype. This scenario is supported by the increased expression of TNFR1 and of genes encoding caspase 1, pro-inflammatory cytokines (IL1β, IL18), chemokines (CCL2, CCL5, CXCL10), and the IFNγ-inducible GBPs GBP2 and GBP5, without concomitant induction of several genes associated with a protective microglia/macrophage phenotype, including CSF1R, MSR1, TREM2, IL10, TGFβ1, and TNFR2. Absence of myelin phagocytic activity in the chronic GM lesions analyzed could explain this finding since, as mentioned above, phagocytosis induces an anti-inflammatory macrophage phenotype (Boven et al., 2006). The present results are in agreement with a recent study showing higher levels of TNFR1, but not TNFR2 RNA in subpial GM lesions from progressive MS cases with prominent meningeal inflammation (Magliozzi et al., 2019). This latter study also shows that TNFR1 induction in subpial GM lesions is associated with increased expression of genes involved in TNFR1-stimulated signaling leading to necroptosis, a different form of necrotic cell death (Magliozzi et al., 2019).

The effect of TNF in regulating inflammation and tissue homeostasis depends on the complex activity of the TNF-TNFR signaling system (Pegoretti et al., 2018). TNF is produced predominantly by macrophages, monocytes and some lymphocyte subsets, including T cells and NK cells; it exists as a membrane bound form that can be released as a soluble form through proteolytic processing (Yang et al., 2018). The two forms of TNF differ in their cellular distribution and ability to activate TNFR1 and TNFR2. TNFR1 is ubiquitously expressed on nearly all cells of the body and can be activated by both membrane and soluble forms of TNF. Conversely, TNFR2 is restricted and inducible in specific cell types, like lymphocytes and monocytes/macrophages, and is fully activated by membrane TNF only (Richter et al., 2012).

Despite we used an enhanced real time RT-PCR to improve detection of low frequency transcripts, TNF RNA was undetectable in all the MS WM and GM parenchymal areas analyzed, while being readily detected in the adjacent perivascular and meningeal immune infiltrates, respectively (Veroni et al., 2018). This finding suggests absence or very low level of TNF transcriptional activity in the MS brain parenchyma and is in agreement with previous gene expression studies of microdissected MS lesions (Koning et al., 2007; Hendrickx et al., 2017; Elkjaer et al., 2019). The fact that TNF is produced by activated macrophages in many pathological conditions and by IFNγ-activated human microglia *in vitro* (Bsibsi et al., 2014) suggests that the CNS microenvironment exerts a tight control on TNF expression in microglia. It is known that GMCSF promotes the differentiation of TNF producing pro-inflammatory macrophages whereas MCSF induces IL10-producing anti-inflammatory macrophages (Verreck et al., 2004). In this study, MCSF RNA was detected in both control and MS WM and GM parenchyma, with no significant differences between control and pathological samples. Conversely, GMCSF RNA was not detected in any of the WM and GM samples analyzed and was undetectable in almost 90% of the CNS immune infiltrates analyzed previously (Veroni et al., 2018). These data suggest that presence of MCSF and lack of GMCSF within the CNS parenchyma may contribute to the generation of an immunosuppressive environment regulating specific features of microglia activation.

Given that the balance between pro- and anti-inflammatory signals may affect the extension and evolution of MS lesions, and that such a balance can be pharmacologically modified, it is important to define the distribution and regulation of molecules involved in CNS healing responses. In this respect, TNFR2 is of particular interest since the specific activation of TNFR2 signaling is highly effective in limiting tissue damage and inflammation and in promoting tissue repair in animal models of neuroinflammation (Brambilla et al., 2011; Taoufik et al., 2011; Williams et al., 2014; Karamita et al., 2017; Steeland et al., 2017) and neurodegeneration (Dong et al., 2016). Moreover, the negative patient outcomes with non-selective anti-TNF therapies (van Oosten et al., 1996; The Lenecercept Multiple Sclerosis Study Group, 1999) suggest that the complete abrogation of TNF signaling has no beneficial effects in MS.

This study shows higher TNFR2 gene expression in the NAWM, actively demyelinating WM lesions and chronic active WM lesion rim adjacent to perivascular cuffs, relatively to non-pathological control WM. Using immunohistochemistry, we show for the first time that TNFR2 expression in the MS brain is restricted to areas of intense microglia/macrophage activation, irrespective of myelin preservation or ongoing myelin damage. Accordingly, TNFR2 was expressed in a substantial proportion of activated microglia in the NAWM, microglia/macrophages at the border of chronic active lesions, and foamy macrophages in actively demyelinating WM lesions. In the MS GM, TNFR2 immunoreactivity was detected mainly in foamy macrophages within active and demyelinating GM lesions, a very rare finding in progressive MS, suggesting higher expression of this receptor on microglia/macrophages during early disease phases when inflammatory cortical demyelination is more prominent (Lucchinetti et al., 2011). In agreement with previous studies (Brambilla et al., 2011), TNFR2 immunoreactivity was not detected in oligodendrocytes. Astrocyte TNFR2 expression in the MS brain was previously shown but localization and frequency of TNFR2+ astrocytes were not clearly defined (Brambilla et al., 2011). In the present study, TNFR2+ astrocytes were observed only at the border of chronic active WM lesions and of some chronic active GM lesions, although this was not a consistent finding. Overall, the present results clearly link TNFR2 expression to microglia/macrophage activation whereas the astrocyte subpopulation expressing TNFR2 in the MS brain remains to be defined.

Since TNF RNA was undetectable in both the MS WM and GM parenchyma, it can be argued that local TNF availability mainly depends on soluble TNF produced by CNS-infiltrating immune cells and diffusing from the perivascular and subarachnoid spaces through the basal lamina and pial membrane, respectively (Magliozzi et al., 2018; Veroni et al., 2018). Because soluble TNF preferentially activates TNFR1 signaling (Grell et al., 1998), it is likely that within the CNS parenchyma TNFR1 would be mainly activated. As it was shown that TNF-TNFR1 signaling induces TNFR2 shedding (Higuchi and Aggarwal, 1994), the increase in TNFR2 expression in microglia/macrophages in the MS brain could be part of a homeostatic response regulating TNF activity, as previously shown for regulatory T cells (van Mierlo et al., 2008). Both the pattern of TNFR2 expression and the tight control of TNF RNA expression in the MS brain parenchyma suggest that a TNF-targeting therapeutic strategy for MS should be oriented toward the development of TNFR2 selective agonists rather than TNFR1 antagonists, as the latter would not be effective in shifting TNF signaling toward TNFR2 in the CNS.

To evaluate the temporal pattern of expression and the functionality of TNFR2 during remyelination, we turned to the model of lysolecithin-induced demyelination in mouse cerebellar slices, which allows to study remyelination as an independent event from demyelination (Doussau et al., 2017). We found that a marked increase in TNFR2, but not TNFR1, RNA level and protein shedding occurs concomitantly with microglia/macrophage activation immediately after myelin damage and during the remyelination phase, suggesting selective and persistent transcriptional activation and translation of TNFR2. Cytokine RNA analysis revealed rapid induction of both pro- (TNF, IL1β) and anti-inflammatory (IL10) cytokines in response to toxin-induced damage. The temporal correlation between TNFR2 and the anti-inflammatory cytokine IL10 is of particular interest as it reveals for the first time a persistent and coordinated transcriptional regulation of these molecules during remyelination and is consistent with the involvement of anti-inflammatory microglia in CNS repair (Miron et al., 2013). The finding that TNF and IL1β RNA were only transiently detected in cerebellar slice cultures (i.e., immediately after toxin removal, but not 2 days later) supports the very tight regulation of pro-inflammatory cytokine expression in the CNS.

This study also shows that selective TNFR2 activation with an agonistic antibody promotes oligodendrocyte maturation in mouse cerebellar slices following toxin-induced myelin damage, as assessed by MBP gene expression and MBP/NFH imaging. These results demonstrate that TNFR2 ligation and activation is an effective means to enhance myelin protein expression and promote a neuroprotective milieu in the CNS. Although evidence has been provided that in the mouse brain TNFR2 is predominantly expressed in microglia (Agresti et al., 1998; Veroni et al., 2010; Zhang et al., 2014), several studies in genetically modified mice suggest that TNFR2 expression in astrocytes and oligodendrocytes might be involved in the stimulation of oligodendrocyte maturation and remyelination (Patel et al., 2012; Fischer et al., 2014; Madsen et al., 2016). Since ablation of microglial TNFR2 was shown to increase demyelination in experimental autoimmune encephalomyelitis (Gao et al., 2017), analysis of cerebellar slices obtained from mice with TNFR2 deletion in microglia should help clarify the contribution of this glial cell population to TNFR2-activated remyelinating pathways.

In conclusion, the results of this study strengthen the concepts that adaptive cytotoxic immunity has a key role in initiating and amplifying detrimental microglia activation and that the balance between dysregulated inflammation and microglia/macrophage-mediated healing responses determines the extent of CNS tissue damage in MS. The findings that TNFR2 is highly expressed in microglia/macrophages in the MS WM both before and during active demyelination and that TNFR2 can be activated by an exogenous compound to promote endogenous myelin formation in an ex-vivo experimental model has translational relevance for MS therapy and may stimulate the development of drugs specifically targeting TNFR2.

## Data Availability Statement

The datasets generated for this study are available on request to the corresponding author.

## Ethics Statement

The studies involving the use of post-mortem human brain tissue were reviewed and approved by the Ethics Committee of Istituto Superiore di Sanità (CE 12/356). The animal study was reviewed and approved by National Center for Animal Research and Welfare of the Istituto Superiore di Sanità and by the Italian Ministry of Health (Authorization 271/SSA/2010).

## Author Contributions

CV and BS contributed to study design, data acquisition analysis and interpretation. BR contributed to data acquisition. CF planned and performed statistical analysis. FA contributed to study design, data analysis and interpretation, and writing of the manuscript. CA contributed to study design, data acquisition, analysis and interpretation, and writing of the manuscript. All authors contributed to the critical review of the manuscript and approved the final version of the manuscript.

## Conflict of Interest

The authors declare that the research was conducted in the absence of any commercial or financial relationships that could be construed as a potential conflict of interest.

## References

[B1] Agrawal S. Anderson P. Durbeej M. van Rooijen N. Ivars F. Opdenakker G.. (2006). Dystroglycan is selectively cleaved at the parenchymal basement membrane at sites of leukocyte extravasation in experimental autoimmune encephalomyelitis. J. Exp. Med. 203, 1007–1019. 10.1084/jem.20051342 16585265 PMC2118280

[B2] Agresti C. Bernardo A. Del Russo N. Marziali G. Battistini A. Aloisi F.. (1998). Synergistic stimulation of MHC class I and IRF-1 gene expression by IFN-gamma and TNF-alpha in oligodendrocytes. Eur. J. Neurosci. 10, 2975–2983. 10.1111/j.1460-9568.1998.00313.x 9758167

[B3] Agresti C. D'Urso D. Levi G. (1996). Reversible inhibitory effects of interferon-gamma and tumour necrosis factor-alpha on oligodendroglial lineage cell proliferation and differentiation *in vitro*. Eur. J. Neurosci. 8, 1106–1116. 10.1111/j.1460-9568.1996.tb01278.x 8752580

[B4] Aloisi F. (2001). Immune function of microglia. Glia. 36, 165–179. 10.1002/glia.1106 11596125

[B5] Angelini D. F. Serafini B. Piras E. Severa M. Coccia E. M. Rosicarelli B.. (2013). Increased CD8+ T cell response to epstein-barr virus lytic antigens in the active phase of multiple sclerosis. PLoS Pathog. 9:e1003220. 10.1371/journal.ppat.1003220 23592979 PMC3623710

[B6] Arango Duque G. Descoteaux A. (2014). Macrophage cytokines: involvement in immunity and infectious diseases. Front. Immunol. 5:491. 10.3389/fimmu.2014.00491 25339958 PMC4188125

[B7] Arnett H. A. Mason J. Marino M. Suzuki K. Matsushima G. K. Ting J. P. (2001). TNF alpha promotes proliferation of oligodendrocyte progenitors and remyelination. Nat. Neurosci. 4, 1116–1122. 10.1038/nn738 11600888

[B8] Babbe H. Roers A. Waisman A. Lassmann H. Goebels N. Hohlfeld R.. (2000). Clonal expansions of CD8(+) T cells dominate the T cell infiltrate in active multiple sclerosis lesions as shown by micromanipulation and single cell polymerase chain reaction. J. Exp. Med. 192, 393–404. 10.1084/jem.192.3.393 10934227 PMC2193223

[B9] Barateiro A. Afonso V. Santos G. Cerqueira J. J. Brites D. van Horssen J.. (2016). S100B as a potential biomarker and therapeutic target in multiple sclerosis. Mol. Neurobiol. 53, 3976–3991. 10.1007/s12035-015-9336-6 26184632

[B10] Becher B. Spath S. Goverman J. (2017). Cytokine networks in neuroinflammation. Nat. Rev. Immunol. 17, 49–59. 10.1038/nri.2016.123 27916979

[B11] Birgbauer E. Rao T. S. Webb M. (2004). Lysolecithin induces demyelination in vitro in a cerebellar slice culture system. J. Neurosci. Res. 78, 157–166. 10.1002/jnr.20248 15378614

[B12] Boven L. A. van Meurs M. van Zwam M. Wierenga-Wolf A. Hintzen R. Q. Boot R. G.. (2006). Myelin-laden macrophages are anti-inflammatory, consistent with foam cells in multiple sclerosis. Brain. 129, 517–526. 10.1093/brain/awh707 16364958

[B13] Brambilla R. Ashbaugh J. J. Magliozzi R. Dellarole A. Karmally S. Szymkowski D. E.. (2011). Inhibition of soluble tumour necrosis factor is therapeutic in experimental autoimmune encephalomyelitis and promotes axon preservation and remyelination. Brain. 134, 2736–2754. 10.1093/brain/awr199 21908877 PMC3170538

[B14] Brenner D. Blaser H. Mak T. W. (2015). Regulation of tumour necrosis factor signalling: Live or let die. Nat. Rev. Immunol. 15, 362–374. 10.1038/nri3834 26008591

[B15] Bsibsi M. Peferoen L. A. Holtman I. R. Nacken P. J. Gerritsen W. H. Witte M. E.. (2014). Demyelination during multiple sclerosis is associated with combined activation of microglia/macrophages by IFN-gamma and alpha B-crystallin. Acta Neuropathol. 128, 215–229. 10.1007/s00401-014-1317-8 24997049

[B16] Butovsky O. Weiner H. L. (2018). Microglial signatures and their role in health and disease. Nat. Rev. Neurosci. 19, 622–635. 10.1038/s41583-018-0057-5 30206328 PMC7255106

[B17] Cantoni C. Bollman B. Licastro D. Xie M. Mikesell R. Schmidt R.. (2015). TREM2 regulates microglial cell activation in response to demyelination *in vivo*. Acta Neuropathol. 129, 429–447. 10.1007/s00401-015-1388-1 25631124 PMC4667728

[B18] Casbon A. J. Long M. E. Dunn K. W. Allen L. A. Dinauer M. C. (2012). Effects of IFN-gamma on intracellular trafficking and activity of macrophage NADPH oxidase flavocytochrome b558. J. Leukoc. Biol. 92, 869–882. 10.1189/jlb.0512244 22822009 PMC3441311

[B19] Colonna M. Butovsky O. (2017). Microglia function in the central nervous system during health and neurodegeneration. Annu. Rev. Immunol. 35, 441–468. 10.1146/annurev-immunol-051116-052358 28226226 PMC8167938

[B20] Denic A. Wootla B. Rodriguez M. (2013). CD8(+) T cells in multiple sclerosis. Expert Opin. Ther. Targets. 17, 1053–1066. 10.1517/14728222.2013.815726 23829711 PMC3928018

[B21] Dong Y. Fischer R. Naude P. J. Maier O. Nyakas C. Duffey M.. (2016). Essential protective role of tumor necrosis factor receptor 2 in neurodegeneration. Proc. Natl. Acad. Sci. U.S.A. 113, 12304–12309. 10.1073/pnas.1605195113 27791020 PMC5087045

[B22] Doussau F. Dupont J. L. Neel D. Schneider A. Poulain B. Bossu J. L. (2017). Organotypic cultures of cerebellar slices as a model to investigate demyelinating disorders. Expert Opin. Drug Discov. 12, 1011–1022. 10.1080/17460441.2017.1356285 28712329

[B23] Dusart I. Airaksinen M. S. Sotelo C. (1997). Purkinje cell survival and axonal regeneration are age dependent: an *in vitro* study. J. Neurosci. 17, 3710–3726. 10.1523/JNEUROSCI.17-10-03710.1997 9133392 PMC6573677

[B24] Eleuteri C. Olla S. Veroni C. Umeton R. Mechelli R. Romano S.. (2017). A staged screening of registered drugs highlights remyelinating drug candidates for clinical trials. Sci. Rep. 7:45780. 10.1038/srep45780 28387380 PMC5384285

[B25] Elkjaer M. L. Frisch T. Reynolds R. Kacprowski T. Burton M. Kruse T. A. (2019). Molecular signature of different lesion types in the brain white matter of patients with progressive multiple sclerosis. Acta Neuropathol. Commun. 7:205 10.1186/s40478-019-0855-7 31829262 PMC6907342

[B26] Fischer M. T. Sharma R. Lim J. L. Haider L. Frischer J. M. Drexhage J.. (2012). NADPH oxidase expression in active multiple sclerosis lesions in relation to oxidative tissue damage and mitochondrial injury. Brain. 135, 886–899. 10.1093/brain/aws012 22366799 PMC3286337

[B27] Fischer M. T. Wimmer I. Hoftberger R. Gerlach S. Haider L. Zrzavy T.. (2013). Disease-specific molecular events in cortical multiple sclerosis lesions. Brain. 136, 1799–1815. 10.1093/brain/awt110 23687122 PMC3673462

[B28] Fischer R. Wajant H. Kontermann R. Pfizenmaier K. Maier O. (2014). Astrocyte-specific activation of TNFR2 promotes oligodendrocyte maturation by secretion of leukemia inhibitory factor. Glia. 62, 272–283. 10.1002/glia.22605 24310780

[B29] Fontaine V. Mohand-Said S. Hanoteau N. Fuchs C. Pfizenmaier K. Eisel U. (2002). Neurodegenerative and neuroprotective effects of tumor necrosis factor (TNF) in retinal ischemia: opposite roles of TNF receptor 1 and TNF receptor 2. J. Neurosci. 22:RC216. 10.1523/JNEUROSCI.22-07-j0001.2002 11917000 PMC6758303

[B30] Gao H. Danzi M. C. Choi C. S. Taherian M. Dalby-Hansen C. Ellman D. G.. (2017). Opposing functions of microglial and macrophagic TNFR2 in the pathogenesis of experimental autoimmune encephalomyelitis. Cell. Rep. 18, 198–212. 10.1016/j.celrep.2016.11.083 28052249 PMC5218601

[B31] Grell M. Wajant H. Zimmermann G. Scheurich P. (1998). The type 1 receptor (CD120a) is the high-affinity receptor for soluble tumor necrosis factor. Proc. Natl. Acad. Sci. U. S. A. 95, 570–575. 10.1073/pnas.95.2.570 9435233 PMC18461

[B32] Griess B. Mir S. Datta K. Teoh-Fitzgerald M. (2020). Scavenging reactive oxygen species selectively inhibits M2 macrophage polarization and their pro-tumorigenic function in part, via Stat3 suppression. Free Radic. Biol. Med. 147, 48–60. 10.1016/j.freeradbiomed.2019.12.018 31863907 PMC10035558

[B33] Gunthner R. Anders H. J. (2013). Interferon-regulatory factors determine macrophage phenotype polarization. Mediators Inflamm. 2013:731023. 10.1155/2013/731023 24379524 PMC3863528

[B34] Haile Y. Simmen K. C. Pasichnyk D. Touret N. Simmen T. Lu J. Q.. (2011). Granule-derived granzyme B mediates the vulnerability of human neurons to T cell-induced neurotoxicity. J. Immunol. 187, 4861–4872. 10.4049/jimmunol.1100943 21964027

[B35] Hanke M. L. Kielian T. (2011). Toll-like receptors in health and disease in the brain: Mechanisms and therapeutic potential. Clin. Sci. (Lond). 121, 367–387. 10.1042/CS20110164 21745188 PMC4231819

[B36] Hendrickx D. A. E. van Scheppingen J. van der Poel M. Bossers K. Schuurman K. G. van Eden C. G.. (2017). Gene expression profiling of multiple sclerosis pathology identifies early patterns of demyelination surrounding chronic active lesions. Front. Immunol. 8:1810. 10.3389/fimmu.2017.01810 29312322 PMC5742619

[B37] Higuchi M. Aggarwal B. B. (1994). TNF induces internalization of the p60 receptor and shedding of the p80 receptor. J. Immunol. 152, 3550–3558. 8144934

[B38] Hodny Z. Reinis M. Hubackova S. Vasicova P. Bartek J. (2015). Interferon gamma/NADPH oxidase defense system in immunity and cancer. Oncoimmunology 5:e1080416. 10.1080/2162402X.2015.1080416 27057461 PMC4801460

[B39] Howell O. W. Reeves C. A. Nicholas R. Carassiti D. Radotra B. Gentleman S. M.. (2011). Meningeal inflammation is widespread and linked to cortical pathology in multiple sclerosis. Brain. 134, 2755–2771. 10.1093/brain/awr182 21840891

[B40] Huang W. C. Sala-Newby G. B. Susana A. Johnson J. L. Newby A. C. (2012). Classical macrophage activation up-regulates several matrix metalloproteinases through mitogen activated protein kinases and nuclear factor-kappaB. PLoS One. 7:e42507. 10.1371/journal.pone.0042507 22880008 PMC3411745

[B41] International Multiple Sclerosis Genetics Consortium (2019). Multiple sclerosis genomic map implicates peripheral immune cells and microglia in susceptibility. Science. 365:aav7188. 10.1126/science.aav7188 31604244 PMC7241648

[B42] Jin L. Raymond D. P. Crabtree T. D. Pelletier S. J. Houlgrave C. W. Pruett T. L.. (2000). Enhanced murine macrophage TNF receptor shedding by cytosine-guanine sequences in oligodeoxynucleotides. J. Immunol. 165, 5153–5160. 10.4049/jimmunol.165.9.5153 11046047

[B43] Karamita M. Barnum C. Mobius W. Tansey M. G. Szymkowski D. E. Lassmann H.. (2017). Therapeutic inhibition of soluble brain TNF promotes remyelination by increasing myelin phagocytosis by microglia. JCI Insight. 2:87455. 10.1172/jci.insight.87455 28422748 PMC5396518

[B44] Kim B. H. Chee J. D. Bradfield C. J. Park E. S. Kumar P. MacMicking J. D. (2016). Interferon-induced guanylate-binding proteins in inflammasome activation and host defense. Nat. Immunol. 17, 481–489. 10.1038/ni.3440 27092805 PMC4961213

[B45] Kim K. S. Park J. Y. Jou I. Park S. M. (2009). Functional implication of BAFF synthesis and release in gangliosides-stimulated microglia. J. Leukoc. Biol. 86, 349–359. 10.1189/jlb.1008659 19406831

[B46] Koning N. Bo L. Hoek R. M. Huitinga I. (2007). Downregulation of macrophage inhibitory molecules in multiple sclerosis lesions. Ann. Neurol. 62, 504–514. 10.1002/ana.21220 17879969

[B47] Krumbholz M. Theil D. Derfuss T. Rosenwald A. Schrader F. Monoranu C. M.. (2005). BAFF is produced by astrocytes and up-regulated in multiple sclerosis lesions and primary central nervous system lymphoma. J. Exp. Med. 201, 195–200. 10.1084/jem.20041674 15642740 PMC2212784

[B48] Kuhlmann T. Ludwin S. Prat A. Antel J. Bruck W. Lassmann H. (2017). An updated histological classification system for multiple sclerosis lesions. Acta Neuropathol. 133, 13–24. 10.1007/s00401-016-1653-y 27988845

[B49] Langlais D. Barreiro L. B. Gros P. (2016). The macrophage IRF8/IRF1 regulome is required for protection against infections and is associated with chronic inflammation. J. Exp. Med. 213, 585–603. 10.1084/jem.20151764 27001747 PMC4821649

[B50] Lassmann H. (2019). Pathogenic mechanisms associated with different clinical courses of multiple sclerosis. Front. Immunol. 9:3116. 10.3389/fimmu.2018.03116 30687321 PMC6335289

[B51] Li Q. Barres B. A. (2018). Microglia and macrophages in brain homeostasis and disease. Nat. Rev. Immunol. 18, 225–242. 10.1038/nri.2017.125 29151590

[B52] Lucchinetti C. F. Popescu B. F. Bunyan R. F. Moll N. M. Roemer S. F. Lassmann H.. (2011). Inflammatory cortical demyelination in early multiple sclerosis. N. Engl. J. Med. 365, 2188–2197. 10.1056/NEJMoa1100648 22150037 PMC3282172

[B53] Machado-Santos J. Saji E. Troscher A. R. Paunovic M. Liblau R. Gabriely G.. (2018). The compartmentalized inflammatory response in the multiple sclerosis brain is composed of tissue-resident CD8+ T lymphocytes and B cells. Brain. 141, 2066–2082. 10.1093/brain/awy151 29873694 PMC6022681

[B54] Madsen P. M. Motti D. Karmally S. Szymkowski D. E. Lambertsen K. L. Bethea J. R.. (2016). Oligodendroglial TNFR2 mediates membrane TNF-dependent repair in experimental autoimmune encephalomyelitis by promoting oligodendrocyte differentiation and remyelination. J. Neurosci. 36, 5128–5143. 10.1523/JNEUROSCI.0211-16.2016 27147664 PMC4854972

[B55] Magliozzi R. Howell O. Vora A. Serafini B. Nicholas R. Puopolo M.. (2007). Meningeal B-cell follicles in secondary progressive multiple sclerosis associate with early onset of disease and severe cortical pathology. Brain. 130, 1089–1104. 10.1093/brain/awm038 17438020

[B56] Magliozzi R. Howell O. W. Durrenberger P. Arico E. James R. Cruciani C.. (2019). Meningeal inflammation changes the balance of TNF signalling in cortical grey matter in multiple sclerosis. J. Neuroinflammation. 16:259. 10.1186/s12974-019-1650-x 31810488 PMC6898969

[B57] Magliozzi R. Howell O. W. Nicholas R. Cruciani C. Castellaro M. Romualdi C.. (2018). Inflammatory intrathecal profiles and cortical damage in multiple sclerosis. Ann. Neurol. 83, 739–755. 10.1002/ana.25197 29518260

[B58] Magliozzi R. Howell O. W. Reeves C. Roncaroli F. Nicholas R. Serafini B.. (2010). A gradient of neuronal loss and meningeal inflammation in multiple sclerosis. Ann. Neurol. 68, 477–493. 10.1002/ana.22230 20976767

[B59] Man S. M. Karki R. Kanneganti T. D. (2017). Molecular mechanisms and functions of pyroptosis, inflammatory caspases and inflammasomes in infectious diseases. Immunol. Rev. 277, 61–75. 10.1111/imr.12534 28462526 PMC5416822

[B60] Marchetti L. Klein M. Schlett K. Pfizenmaier K. Eisel U. L. (2004). Tumor necrosis factor (TNF)-mediated neuroprotection against glutamate-induced excitotoxicity is enhanced by N-methyl-D-aspartate receptor activation. essential role of a TNF receptor 2-mediated phosphatidylinositol 3-kinase-dependent NF-kappa B pathway. J. Biol. Chem. 279, 32869–32881. 10.1074/jbc.M311766200 15155767

[B61] Martinez F. O. Gordon S. Locati M. Mantovani A. (2006). Transcriptional profiling of the human monocyte-to-macrophage differentiation and polarization: new molecules and patterns of gene expression. J. Immunol. 177, 7303–7311. 10.4049/jimmunol.177.10.7303 17082649

[B62] Miron V. E. Boyd A. Zhao J. W. Yuen T. J. Ruckh J. M. Shadrach J. L.. (2013). M2 microglia and macrophages drive oligodendrocyte differentiation during CNS remyelination. Nat. Neurosci. 16, 1211–1218. 10.1038/nn.3469 23872599 PMC3977045

[B63] Nathan C. F. Murray H. W. Wiebe M. E. Rubin B. Y. (1983). Identification of interferon-gamma as the lymphokine that activates human macrophage oxidative metabolism and antimicrobial activity. J. Exp. Med. 158, 670–689. 10.1084/jem.158.3.670 6411853 PMC2187114

[B64] Patel J. R. Williams J. L. Muccigrosso M. M. Liu L. Sun T. Rubin J. B.. (2012). Astrocyte TNFR2 is required for CXCL12-mediated regulation of oligodendrocyte progenitor proliferation and differentiation within the adult CNS. Acta Neuropathol. 124, 847–860. 10.1007/s00401-012-1034-0 22933014 PMC3508279

[B65] Pegoretti V. Baron W. Laman J. D. Eisel U. L. M. (2018). Selective modulation of TNF-TNFRs signaling: Insights for multiple sclerosis treatment. Front. Immunol. 9:925. 10.3389/fimmu.2018.00925 29760711 PMC5936749

[B66] Pender M. P. Burrows S. R. (2014). Epstein-barr virus and multiple sclerosis: Potential opportunities for immunotherapy. Clin. Transl. Immunol. 3:e27. 10.1038/cti.2014.25 25505955 PMC4237030

[B67] Platanitis E. Decker T. (2018). Regulatory networks involving STATs, IRFs, and NFkappaB in inflammation. Front. Immunol. 9:2542. 10.3389/fimmu.2018.02542 30483250 PMC6242948

[B68] PrabhuDas M. R. Baldwin C. L. Bollyky P. L. Bowdish D. M. E. Drickamer K. Febbraio M.. (2017). A consensus definitive classification of scavenger receptors and their roles in health and disease. J. Immunol. 198, 3775–3789. 10.4049/jimmunol.1700373 28483986 PMC5671342

[B69] Probert L. (2015). TNF and its receptors in the CNS: The essential, the desirable and the deleterious effects. Neuroscience 302, 2–22. 10.1016/j.neuroscience.2015.06.038 26117714

[B70] Reich D. S. Lucchinetti C. F. Calabresi P. A. (2018). Multiple sclerosis. N. Engl. J. Med. 378, 169–180. 10.1056/NEJMra1401483 29320652 PMC6942519

[B71] Richter C. Messerschmidt S. Holeiter G. Tepperink J. Osswald S. Zappe A.. (2012). The tumor necrosis factor receptor stalk regions define responsiveness to soluble versus membrane-bound ligand. Mol. Cell. Biol. 32, 2515–2529. 10.1128/MCB.06458-11 22547679 PMC3434479

[B72] Ruytinx P. Proost P. van Damme J. Struyf S. (2018). Chemokine-induced macrophage polarization in inflammatory conditions. Front. Immunol. 9:1930. 10.3389/fimmu.2018.01930 30245686 PMC6137099

[B73] Schuh C. Wimmer I. Hametner S. Haider L. Van Dam A. M. Liblau R. S.. (2014). Oxidative tissue injury in multiple sclerosis is only partly reflected in experimental disease models. Acta Neuropathol. 128, 247–266. 10.1007/s00401-014-1263-5 24622774 PMC4102830

[B74] Serafini B. Rosicarelli B. Franciotta D. Magliozzi R. Reynolds R. Cinque P.. (2007). Dysregulated epstein-barr virus infection in the multiple sclerosis brain. J. Exp. Med. 204, 2899–2912. 10.1084/jem.20071030 17984305 PMC2118531

[B75] Serafini B. Rosicarelli B. Magliozzi R. Stigliano E. Aloisi F. (2004). Detection of ectopic B-cell follicles with germinal centers in the meninges of patients with secondary progressive multiple sclerosis. Brain Pathol. 14, 164–174. 10.1111/j.1750-3639.2004.tb00049.x 15193029 PMC8095922

[B76] Serafini B. Rosicarelli B. Magliozzi R. Stigliano E. Capello E. Mancardi G. L.. (2006). Dendritic cells in multiple sclerosis lesions: Maturation stage, myelin uptake, and interaction with proliferating T cells. J. Neuropathol. Exp. Neurol. 65, 124–141. 10.1093/jnen/65.2.124 16462204

[B77] Serafini B. Rosicarelli B. Veroni C. Mazzola G. A. Aloisi F. (2019). Epstein-barr virus-specific CD8 T cells selectively infiltrate the brain in multiple sclerosis and interact locally with virus-infected cells: Clue for a virus-driven immunopathological mechanism. J. Virol. 93:e00980-19. 10.1128/JVI.00980-19 31578295 PMC6880158

[B78] Serafini B. Severa M. Columba-Cabezas S. Rosicarelli B. Veroni C. Chiappetta G.. (2010). Epstein-barr virus latent infection and BAFF expression in B cells in the multiple sclerosis brain: implications for viral persistence and intrathecal B-cell activation. J. Neuropathol. Exp. Neurol. 69, 677–693. 10.1097/NEN.0b013e3181e332ec 20535037

[B79] Sobottka B. Harrer M. D. Ziegler U. Fischer K. Wiendl H. Hunig T.. (2009). Collateral bystander damage by myelin-directed CD8+ T cells causes axonal loss. Am. J. Pathol. 175, 1160–1166. 10.2353/ajpath.2009.090340 19700745 PMC2731134

[B80] Steeland S. van Ryckeghem S. van Imschoot G. de Rycke R. Toussaint W. Vanhoutte L.. (2017). TNFR1 inhibition with a nanobody protects against EAE development in mice. Sci. Rep. 7:13984. 10.1038/s41598-017-13984-y 29057962 PMC5651799

[B81] Suvannavejh G. C. Lee H. O. Padilla J. Dal Canto M. C. Barrett T. A. Miller S. D. (2000). Divergent roles for p55 and p75 tumor necrosis factor receptors in the pathogenesis of MOG(35-55)-induced experimental autoimmune encephalomyelitis. Cell. Immunol. 205, 24–33. 10.1006/cimm.2000.1706 11078604

[B82] Taoufik E. Tseveleki V. Chu S. Y. Tselios T. Karin M. Lassmann H.. (2011). Transmembrane tumour necrosis factor is neuroprotective and regulates experimental autoimmune encephalomyelitis via neuronal nuclear factor-kappaB. Brain 134, 2722–2735. 10.1093/brain/awr203 21908876

[B83] Taylor G. S. Long H. M. Brooks J. M. Rickinson A. B. Hislop A. D. (2015). The immunology of Epstein-Barr virus-induced disease. Annu. Rev. Immunol. 33, 787–821. 10.1146/annurev-immunol-032414-112326 25706097

[B84] The Lenecercept Multiple Sclerosis Study Group. (1999). TNF neutralization in MS: results of a randomized, placebo-controlled multicenter study. The Lenercept Multiple Sclerosis study group and the University of British Columbia MS/MRI analysis group. Neurology. 53, 457–465. 10449104

[B85] Tretina K. Park E. S. Maminska A. MacMicking J. D. (2019). Interferon-induced guanylate-binding proteins: guardians of host defense in health and disease. J. Exp. Med. 216, 482–500. 10.1084/jem.20182031 30755454 PMC6400534

[B86] van der Poel M. Ulas T. Mizee M. R. Hsiao C. C. Miedema S. S. M. Adelia. (2019). Transcriptional profiling of human microglia reveals grey-white matter heterogeneity and multiple sclerosis-associated changes. Nat. Commun. 10:1139. 10.1038/s41467-019-08976-7 30867424 PMC6416318

[B87] van Mierlo G. J. Scherer H. U. Hameetman M. Morgan M. E. Flierman R. Huizinga T. W.. (2008). Cutting edge: TNFR-shedding by CD4+CD25+ regulatory T cells inhibits the induction of inflammatory mediators. J. Immunol. 180, 2747–2751. 10.4049/jimmunol.180.5.2747 18292492

[B88] van Nierop G. P. van Luijn M. M. Michels S. S. Melief M. J. Janssen M. Langerak A. W.. (2017). Phenotypic and functional characterization of T cells in white matter lesions of multiple sclerosis patients. Acta Neuropathol. 134, 383–401. 10.1007/s00401-017-1744-4 28624961 PMC5563341

[B89] van Oosten B. W. Barkhof F. Truyen L. Boringa J. B. Bertelsmann F. W. von Blomberg B. M.. (1996). Increased MRI activity and immune activation in two multiple sclerosis patients treated with the monoclonal anti-tumor necrosis factor antibody cA2. Neurology. 47, 1531–1534. 10.1212/WNL.47.6.1531 8960740

[B90] Veroni C. Gabriele L. Canini I. Castiello L. Coccia E. Remoli M. E.. (2010). Activation of TNF receptor 2 in microglia promotes induction of anti-inflammatory pathways. Mol. Cell. Neurosci. 45, 234–244. 10.1016/j.mcn.2010.06.014 20600925

[B91] Veroni C. Serafini B. Rosicarelli B. Fagnani C. Aloisi F. (2018). Transcriptional profile and epstein-barr virus infection status of laser-cut immune infiltrates from the brain of patients with progressive multiple sclerosis. J. Neuroinflammation. 15:18. 10.1186/s12974-017-1049-5 29338732 PMC5771146

[B92] Verreck F. A. de Boer T. Langenberg D. M. Hoeve M. A. Kramer M. Vaisberg E.. (2004). Human IL-23-producing type 1 macrophages promote but IL-10-producing type 2 macrophages subvert immunity to (myco)bacteria. Proc. Natl. Acad. Sci. U.S.A. 101, 4560–4565. 10.1073/pnas.0400983101 15070757 PMC384786

[B93] Vilhardt F. Haslund-Vinding J. Jaquet V. McBean G. (2017). Microglia antioxidant systems and redox signalling. Br. J. Pharmacol. 174, 1719–1732. 10.1111/bph.13426 26754582 PMC5446583

[B94] Viola A. Munari F. Sanchez-Rodriguez R. Scolaro T. Castegna A. (2019). The metabolic signature of macrophage responses. Front. Immunol. 10:1462. 10.3389/fimmu.2019.01462 31333642 PMC6618143

[B95] Williams S. K. Maier O. Fischer R. Fairless R. Hochmeister S. Stojic A.. (2014). Antibody-mediated inhibition of TNFR1 attenuates disease in a mouse model of multiple sclerosis. PLoS One. 9:e90117. 10.1371/journal.pone.0090117 24587232 PMC3938650

[B96] Willis S. N. Stathopoulos P. Chastre A. Compton S. D. Hafler D. A. O'Connor K. C. (2015). Investigating the antigen specificity of multiple sclerosis central nervous system-derived immunoglobulins. Front. Immunol. 6:600. 10.3389/fimmu.2015.00600 26648933 PMC4663633

[B97] Yang S. Wang J. Brand D. D. Zheng S. G. (2018). Role of TNF-TNF receptor 2 signal in regulatory T cells and its therapeutic implications. Front. Immunol. 9:784. 10.3389/fimmu.2018.00784 29725328 PMC5916970

[B98] Zeis T. Graumann U. Reynolds R. Schaeren-Wiemers N. (2008). Normal-appearing white matter in multiple sclerosis is in a subtle balance between inflammation and neuroprotection. Brain 131, 288–303. 10.1093/brain/awm291 18056737

[B99] Zhang Y. Chen K. Sloan S. A. Bennett M. L. Scholze A. R. O'Keeffe S.. (2014). An RNA-sequencing transcriptome and splicing database of glia, neurons, and vascular cells of the cerebral cortex. J. Neurosci. 34, 11929–11947. 10.1523/JNEUROSCI.1860-14.2014 25186741 PMC4152602

[B100] Zhang Z. Zhang Z. Y. Schittenhelm J. Wu Y. Meyermann R. Schluesener H. J. (2011). Parenchymal accumulation of CD163+ macrophages/microglia in multiple sclerosis brains. J. Neuroimmunol. 237, 73–79. 10.1016/j.jneuroim.2011.06.006 21737148

[B101] Zrzavy T. Hametner S. Wimmer I. Butovsky O. Weiner H. L. Lassmann H. (2017). Loss of ‘homeostatic’ microglia and patterns of their activation in active multiple sclerosis. Brain 140, 1900–1913. 10.1093/brain/awx113 28541408 PMC6057548

