# Supplementary material for: Connecting Immune Cell Infiltration to the Multitasking Microglia Response and TNF Receptor 2 Induction in the Multiple Sclerosis Brain
Source: Front Cell Neurosci. 2020 Jul 7;14:190. doi: 10.3389/fncel.2020.00190 (PMC7359043; doi:10.3389/fncel.2020.00190)
Supplement: Supplementary file 1 [file Table_1.pdf]

Supplementary Table 1. List of Taqman human inventoried and EBV self-designed assays used to study cellular and viral gene expression

Gene	Assay code	Gene	Assay code
Glyceraldehyde 3-phosphate dehydrogenase (GAPDH)	Hs99999905_m1	CCR2	Hs00704702_s1
Cathepsin S	Hs00175407_m1	C-X-C motif chemokine 10 (CXCL10)	Hs00171042_m1
HLA-DRA or major histocompatibility complex (MHC) class II	Hs00219575_m1	CXCL16	Hs00222859_m1
MHC class II transactivator (CIITA)	Hs00172106_m1	C-X3-C motif chemokine receptor 1 (CX3CR1)	Hs01922583_s1
Regulatory factor X5 (RFX5)	Hs00230841_m1	Inducible nitric oxide synthase (iNOS)	Hs01075529_m1
CD86	Hs01567026_m1	CYBB	Hs00166163_m1
CD40	Hs01002915_g1	CYBA	Hs00609145_m1
Runt related transcription factor 1 (RUNX1)	Hs02558380_s1	Glutathione peroxidase 1 (GPX1)	Hs00829989_gH
Interferon regulatory factor 1 (IRF1)	Hs00971965_m1	Heme oxygenase 1 (HMOX1)	Hs01110250_m1
IRF4	Hs00180031_m1	Nuclear factor erythroid 2-related factor 2 (NRF2)	Hs00975961_g1
IRF8	Hs00175238_m1	Metalloproteinase 1 (MMP1)	Hs00899658_m1
Transmembrane protein 119 (TMEM119)	Hs01938722_u1	MMP2	Hs01548727_m1
Purinergic receptor P2RY12	Hs01881698_s1	MMP9	Hs00234579_m1
Colony stimulating factor 1 receptor (CSF1R)	Hs00911250_m1	Toll-like receptor 2 (TLR2)	Hs02621280_s1
Triggering receptor expressed on myeloid cells 2 (TREM2)	Hs00219132_m1	TLR3	Hs01551078_m1
CD68	Hs00154355_m1	TLR7	Hs01933259_s1
Macrophage scavenger receptor 1 (MSR1)	Hs00234007_m1	TLR9	Hs00152973_m1
Mannose receptor-c Type 1 (MRC1)	Hs00267207_m1	Guanylate binding protein 1 (GBP1)	Hs00977005_m1
CD163	Hs00174705_m1	GBP2	Hs00894837_m1
Caspase 1	Hs00354836_m1	GBP4	Hs00925073_m1
NLR family pyrin domain containing 3 (NLRP3)	Hs00918082_m1	GBP5	Hs00369472_m1
Cyclooxygenase 2 (COX2)	Hs00153133_m1	Interferon β (IFNβ)	Hs01077958_s1
Tumor necrosis factor (TNF)	Hs00174128_m1	IFNγ	Hs00174143_m1
TNF receptor 1 (TNFR1)	Hs01042313_m1	IFNγ receptor 1 (IFNγR1)	Hs00988304_m1
TNFR2	Hs00153550_m1	Janus kinase 2 (JAK2)	Hs01078136_m1
Interleukin 1α (IL1α)	Hs00174092_m1	Signal transducer and activator of transcription 1 (STAT1)	Hs01013996_m1
IL1β	Hs01555410_m1	STAT2	Hs01013115_g1
IL6	Hs00985639_m1	Interferon induced protein with tetratricopeptide repeats 1 (IFIT1)	Hs01675197_m1
IL10	Hs00961622_m1	Interferon alpha inducible protein 6 (IFI6)	Hs00242571_m1
IL16	Hs00189606_m1	IFI16	Hs00986757_m1
IL18	Hs01038788_m1	Myxovirus resistance protein (MxA)	Hs00895608_m1
B cell activating factor (BAFF)	Hs00198106_m1	2'-5'-oligoadenylate synthetase 1 (OAS1)	Hs00169345_m1
Transforming growth factor β1 (TGFβ1)	Hs00998133_m1	OAS2	Hs00942643_m1
Granulocyte-macrophage colony stimulating factor (GMCSF)	Hs00929873_m1	Fas ligand	Hs00181226_g1
Macrophage colony stimulating factor (MCSF)/CSF1	Hs00174164_m1	CD8	Hs00233520_m1
Secreted phosphoprotein 1 (SPP1)	Hs00959010_m1	CD20	Hs00544818_m1
Chemokine ligand 2 (CCL2)	Hs00234140_m1	CD138	Hs00896423_m1
CCL4	Hs99999148_m1	Myelin basic protein (MBP)	Hs00921945_m1
CCL5	Hs00982282_m1	Glial fibrillary acidic protein (GFAP)	Hs00909233_m1
C-C chemokine receptor type 1 (CCR1)	Hs00928897_s1		

Gene	Forward primer	Reverse primer	FAM-labelled probe
EBV-encoded small RNA (EBER) 1	GTTGCCCTAGTGGTTTCG	CCCCGGGACTTGACC	ACACACCGCCAACGCTCAGT
EBV latent membrane protein (LMP) 1	GGACAACGACACAGTGATG AACA	CATCGGTAGCTTGTTGAGGGT	CCACCACGATGACTCC
