# Supplementary material for: Connecting Immune Cell Infiltration to the Multitasking Microglia Response and TNF Receptor 2 Induction in the Multiple Sclerosis Brain
Source: Front Cell Neurosci. 2020 Jul 7;14:190. doi: 10.3389/fncel.2020.00190 (PMC7359043; doi:10.3389/fncel.2020.00190)
Supplement: Supplementary file 2 [file Table_2.pdf]

Supplementary Table 2. Expression level and frequency of the 71 immune response-related genes analyzed in white matter and grey matter parenchymal areas isolated from control and MS brains.

Gene expression values are presented as $2^{-\Delta Ct}$ relative to GAPDH; mean values ± SD are shown. Fold changes in gene expression values in MS WM and GM samples compared to control WM and GM, respectively, are shown in bold. n.d = not defined; fold change was not defined because gene expression was undetectable in control or WM samples. Percentages represent the fraction of microdissected samples from each brain parenchymal area with detectable gene expression.

Function	Gene	Control WM n=5	NAWM n=14	Active WM lesions n=13	Chronic active WM lesion rim n=8	Control GM n = 6	Subpial GM lesions n=24
		Mean ± SD<br>Frequency (%)	Mean ± SD<br>**Fold change**<br>Frequency (%)	Mean ± SD<br>**Fold change**<br>Frequency (%)	Mean ± SD<br>**Fold change**<br>Frequency (%)	Mean ± SD<br>Frequency (%)	Mean ± SD<br>**Fold change**<br>Frequency (%)
Microglia-specific molecules	TMEM119	0.008±0.02<br>20%	0.03±0.08<br>**4.3**<br>36%	0.11±0.23<br>**14**<br>46%	0.03±0.07<br>**4.3**<br>25%	Undetectable	0.04±0.10<br>n.d.<br>50%
	P2RY12	0.02±0.04<br>60%	0.04±0.07<br>**2**<br>43%	0.13±0.22<br>**6.5**<br>69%	0.04±0.06<br>**2**<br>38%	0.05±0.06<br>50%	0.07±0.12<br>**1.4**<br>58%
Microglia/ macrophage signaling molecules	TREM2	0.000005±0.0001<br>20%	0.05±0.11<br>**10.000**<br>79%	0.04±0.05<br>**8.000**<br>77%	0.06±0.14<br>**12.000**<br>88%	0.013±0.03<br>33%	0.006±0.01<br>**0.5**<br>54%
	CSF1R	0.01±0.01<br>60%	0.045±0.04<br>**4.5**<br>93%	0.042±0.03<br>**4.2**<br>92%	0.037±0.04<br>**3.7**<br>88%	0.03±0.04<br>67%	0.02±0.03<br>**0.8**<br>92%
Transcription factors	RUNX1	Undetectable	0.03±0.09<br>n.d.<br>21%	0.17±0.26<br>n.d.<br>54%	0.25±0.61<br>n.d.<br>38%	0.007±0.017<br>17%	0.02±0.03<br>**2.9**<br>42%
	IRF1	0.013±0.029<br>20%	0.08±0.08<br>**6.2**<br>79%	0.11±0.12<br>**8.5**<br>77%	0.12±0.21<br>**9.2**<br>38%	0.05±0.07<br>50%	0.07±0.18<br>**1.4**<br>63%
	IRF4	Undetectable	0.07±0.15<br>n.d.<br>64%	0.47±0.64<br>n.d.<br>85%	0.71±1.35<br>n.d.<br>63%	0.014±0.032<br>17%	0.18±0.51<br>**12.9**<br>46%
	IRF8	0.0016±0.004<br>20%	0.08±0.07<br>**50**<br>79%	0.22±0.32<br>**138**<br>69%	0.19±0.21<br>**119**<br>75%	0.05±0.06<br>50%	0.19±0.24<br>**3.8**<br>79%
IFN-pathway activation	IFNβ	0.00055±0.001<br>20%	0.027±0.065<br>**49**<br>36%	0.13±0.24<br>**241**<br>62%	0.028±0.05<br>**51**<br>38%	Undetectable	0.03±0.08<br>n.d.<br>46%
	IFNγR1	0.049±0.1<br>60%	0.034±0.045<br>**0.7**<br>64%	0.06±0.08<br>**1.3**<br>77%	0.09±0.14<br>**1.7**<br>50%	0.003±0.004<br>33%	0.02±0.04<br>**8.9**<br>54%
	JAK2	0.024±0.03<br>60%	0.032±0.032<br>**1.3**<br>71%	0.05±0.05<br>**2.2**<br>85%	0.07±0.1<br>**2.9**<br>63%	0.05±0.04<br>83%	0.07±0.09<br>**1.5**<br>79%
	STAT1	0.06±0.058<br>60%	0.12±0.16<br>**2.0**<br>64%	0.25±0.15<br>**4.1**<br>92%	0.18±0.27<br>**3.0**<br>63%	0.1±0.12<br>67%	0.37±0.52<br>**3.6**<br>83%
	STAT2	0.27±0.25<br>80%	0.35±0.39<br>**1.3**<br>64%	0.73±0.6<br>**2.7**<br>92%	1.2±1.2<br>**4.5**<br>88%	0.2±0.19<br>83%	0.38±0.42<br>**1.9**<br>92%
	IFI6	0.01±0.02<br>40%	0.03±0.042<br>**3.0**<br>71%	0.07±0.08<br>**7**<br>77%	0.06±0.11<br>**6**<br>50%	0.067±0.059<br>83%	0.1±0.16<br>**1.5**<br>75%

	IFI16	0.066±0.09<br><br>80%	0.19±0.17<br>**3.0**<br>93%	0.41±0.23<br>**6.2**<br>100%	0.55±0.45<br>**8.3**<br>100%	0.049±0.035<br><br>83%	0.28±0.22<br>**5.7**<br>96%
	IFIT1	0.003±0.007<br><br>20%	Undetectable<br><br>n.d.	0.0015±0.005<br>**0.5**<br>8%	0.013±0.04<br>**4.1**<br>13%	0.002±0.004<br><br>33%	0.01±0.02<br>**5.0**<br>29%
	MXA	0.03±0.03<br><br>60%	0.08±0.07<br>**2.5**<br>93%	0.18±0.13<br>**5.4**<br>92%	0.11±0.09<br>**3.3**<br>75%	0.09±0.09<br><br>83%	0.09±0.09<br>1.0<br>83%
	OAS1	0.003±0.01<br><br>40%	0.01±0.014<br>**3.4**<br>71%	0.019±0.017<br>**6.8**<br>77%	0.024±0.05<br>**8.3**<br>63%	0.023±0.042<br><br>67%	0.004±0.01<br>**0.2**<br>50%
	OAS2	0.02±0.05<br><br>20%	0.02±0.04<br>**1.0**<br>50%	0.1±0.12<br>**5**<br>77%	0.035±0.06<br>**1.8**<br>50%	0.006±0.014<br><br>17%	0.03±0.07<br>**5.0**<br>54%
Antigen presentation	Cathepsin S	0.019±0.04<br><br>20%	0.42±0.3<br>**22.2**<br>100%	0.93±0.87<br>**49**<br>92%	0.9±1.05<br>**47.8**<br>88%	0.06±0.07<br><br>50%	0.33±0.44<br>**5.5**<br>83%
	CIITA	0.012±0.02<br><br>60%	0.03±0.02<br>**2.6**<br>100%	0.04±0.04<br>**3.3**<br>92%	0.13±0.18<br>**10.8**<br>88%	0.004±0.008<br><br>33%	0.04±0.06<br>**10**<br>79%
	RFX5	0.0002±0.0005<br><br>20%	0.04±0.06<br>**200**<br>64%	0.06±0.06<br>**300**<br>77%	0.02±0.03<br>**100**<br>38%	0.018±0.03<br><br>50%	0.1±0.22<br>**5.6**<br>71%
	HLA-DRA	0.03±0.02<br><br>100%	0.42±0.42<br>**14.2**<br>100%	0.57±0.5<br>**19.2**<br>100%	0.63±0.71<br>**21.2**<br>100%	0.027±0.018<br><br>83%	0.29±0.26<br>**10.9**<br>100%
T-cell costimulation	CD86	Undetectable	0.02±0.02<br>n.d.<br>57%	0.04±0.05<br>n.d.<br>69%	0.01±0.02<br>n.d.<br>38%	0.002±0.004<br><br>17%	0.012±0.03<br>**6.0**<br>50%
Antigen presenting cell stimulation	CD40	Undetectable	0.04±0.05<br>n.d.<br>71%	0.05±0.08<br>n.d.<br>62%	0.18±0.04<br>n.d.<br>38%	0.001±0.002<br><br>17%	0.025±0.04<br>**25**<br>54%
Scavenger receptors/ phagocytosis	CD68	0.054±0.06<br><br>80%	0.66±0.43<br>**12**<br>100%	1.35±0.96<br>**25**<br>100%	1.47±2.0<br>**27**<br>100%	0.09±0.14<br><br>50%	0.13±0.08<br>**1.4**<br>100%
	MSR1	0.002±0.003<br><br>40%	0.17±0.15<br>**85**<br>93%	0.53±0.57<br>**265**<br>92%	0.42±0.55<br>**210**<br>88%	0.018±0.041<br><br>17%	0.06±0.11<br>**3.3**<br>58%
	MRC1	0.000022±0.00005<br><br>20%	0.0009±0.002<br>**41**<br>29%	0.0015±0.003<br>**68**<br>46%	0.0017±0.002<br>**77**<br>50%	0.002±0.005<br><br>17%	0.013±0.06<br>**6.5**<br>42%
	CD163	0.09±0.18<br><br>60%	0.07±0.13<br>**0.8**<br>36%	1.11±1.46<br>**12**<br>85%	1.47±2.3<br>**15.6**<br>75%	Undetectable	0.07±0.10<br>n.d.<br>54%
	CXCL16	0.17±0.24<br><br>100%	0.25±0.14<br>**1.5**<br>100%	0.73±0.81<br>**4.3**<br>100%	0.51±0.62<br>**3**<br>100%	0.08±0.07<br><br>83%	0.18±0.16<br>**2.1**<br>100%
Inflammation	Nlrp3	0.035±0.08<br><br>20%	0.005±0.01<br>**0.15**<br>29%	0.023±0.02<br>**0.7**<br>62%	0.022±0.03<br>**0.6**<br>50%	0.028±0.051<br><br>50%	0.025±0.07<br>**0.9**<br>38%
	Caspase 1	Undetectable	0.07±0.06<br>n.d.<br>86%	0.13±0.16<br>n.d.<br>100%	0.42±0.69<br>n.d.<br>88%	0.007±0.01<br><br>33%	0.11±0.13<br>**15.7**<br>88%

	COX2	0.0028±0.006<br><br>20%	0.011±0.027<br>**3.9**<br>21%	0.028±0.04<br>**10**<br>46%	0.16±0.33<br>**57**<br>50%	0.0021±0.005<br><br>17%	0.0027±0.007<br>**1.3**<br>21%
IFNγ-inducible guanylate binding proteins/ antimicrobial function	GBP1	0.002±0.005<br><br>20%	0.086±0.18<br>**43**<br>36%	0.091±0.12<br>**45**<br>69%	0.095±0.17<br>**47**<br>63%	0.026±0.06<br><br>17%	0.087±0.24<br>**3.4**<br>54%
	GBP2	0.011±0.025<br><br>20%	0.11±0.1<br>**9.5**<br>93%	0.17±0.15<br>**15**<br>85%	0.3±0.35<br>**27**<br>88%	0.005±0.008<br><br>33%	0.11±0.17<br>**21**<br>71%
	GBP4	Undetectable	0.005±0.01<br>n.d.<br>36%	0.001±0.003<br>n.d.<br>15%	0.02±0.05<br>n.d.<br>38%	0.00001±0.00003<br><br>17%	0.0017±0.004<br>**135**<br>38%
	GBP5	Undetectable	0.009±0.021<br>n.d.<br>29%	0.023±0.04<br>n.d.<br>46%	0.01±0.01<br>n.d.<br>25%	Undetectable	0.02±0.05<br>n.d.<br>38%
Cytokines/ receptors	TNF	Undetectable	Undetectable	Undetectable	Undetectable	Undetectable	Undetectable
	TNFR1	0.004±0.01<br><br>40%	0.03±0.05<br>**8**<br>79%	0.03±0.02<br>**8**<br>100%	0.02±0.04<br>**5.6**<br>63%	0.002±0.004<br><br>33%	0.06±0.16<br>**3.0**<br>75%
	TNFR2	0.0076±0.01<br><br>60%	0.077±0.06<br>**10**<br>100%	0.12±0.12<br>**15.8**<br>92%	0.11±0.14<br>**14.5**<br>88%	0.011±0.021<br><br>33%	0.05±0.1<br>**4.6**<br>71%
	IL1α	0.00057±0.001<br><br>20%	0.008±0.017<br>**14**<br>36%	0.0032±0.012<br>**5.6**<br>8%	Undetectable	Undetectable	0.0002±0.0008<br>n.d.<br>8%
	IL1β	0.013±0.02<br><br>40%	0.06±0.12<br>**4.6**<br>50%	0.05±0.06<br>**3.8**<br>77%	0.26±0.69<br>**20**<br>50%	0.0048±0.011<br><br>17%	0.1±0.15<br>**22**<br>71%
	IL6	0.013±0.03<br>40%	Undetectable	0.026±0.05<br>**2**<br>38%	0.0006±0.002<br>**0.05**<br>13%	0.0018±0.004<br><br>17%	0.0088±0.023<br>**4.7**<br>33%
	IL10	Undetectable	0.02±0.02<br>n.d.<br>71%	0.03±0.04<br>n.d.<br>69%	0.01±0.03<br>n.d.<br>25%	0.01±0.022<br><br>17%	0.003±0.007<br>**0.3**<br>25%
	IL16	0.0055±0.012<br><br>20%	0.046±0.07<br>**8.2**<br>64%	0.054±0.05<br>**9.6**<br>69%	0.047±0.09<br>**8.4**<br>38%	0.0037±0.008<br>17%	0.014±0.019<br>**3.7**<br>58%
	IL18	Undetectable	0.004±0.01<br>n.d.<br>43%	0.005±0.01<br>n.d.<br>23%	0.002±0.003<br>n.d.<br>25%	Undetectable	0.012±0.040<br>n.d.<br>38%
	BAFF	0.0017±0.004<br><br>20%	0.009±0.02<br>**5.3**<br>43%	0.1±0.18<br>**59**<br>85%	0.02±0.03<br>**12**<br>50%	Undetectable	0.007±0.009<br>n.d.<br>46%
	TGFβ1	0.065±0.09<br><br>60%	0.22±0.17<br>**3.3**<br>93%	0.48±0.5<br>**7.3**<br>92%	0.5±0.53<br>**7.6**<br>100%	0.09±0.09<br><br>67%	0.18±0.21<br>**2.0**<br>83%
	GMCSF	Undetectable	Undetectable	Undetectable	Undetectable	Undetectable	Undetectable
	MCSF/CSF1	0.075±0.08<br><br>80%	0.048±0.059<br>**0.6**<br>50%	0.12±0.13<br>**1.6**<br>77%	0.045±0.066<br>**0.6**<br>38%	0.04±0.06<br><br>33%	0.06±0.09<br>**1.5**<br>67%
	SPP1	7.6±7.0<br><br>100%	10±14<br>**1.3**<br>100%	27±35<br>**3.5**<br>100%	13.4±12.1<br>**1.8**<br>100%	2.7±4.1<br><br>100%	3.4±3.3<br>**1.3**<br>100%

Chemokines/ receptors	CCL2	0.004±0.007<br>40%	0.04±0.05<br>**10**<br>57%	0.03±0.04<br>**8.2**<br>62%	0.05±0.09<br>**13.7**<br>50%	Undetectable	0.03±0.05<br>n.d.<br>54%
	CCL4	0.00039±0.001<br>20%	0.0077±0.016<br>**19.8**<br>29%	0.008±0.016<br>**20.4**<br>23%	Undetectable	Undetectable	0.001±0.005<br>n.d.<br>8%
	CCL5	Undetectable	0.14±0.28<br>n.d.<br>57%	0.1±0.11<br>n.d.<br>69%	0.23±0.41<br>n.d.<br>50%	Undetectable	0.07±0.16<br>n.d.<br>38%
	CXCL10	Undetectable	0.02±0.05<br>n.d.<br>36%	0.01±0.02<br>n.d.<br>31%	0.01±0.01<br>n.d.<br>38%	Undetectable	0.02±0.04<br>n.d.<br>38%
	CCR1	Undetectable	0.004±0.008<br>n.d.<br>29%	0.035±0.04<br>n.d.<br>77%	0.05±0.07<br>n.d.<br>63%	0.00026±0.0006<br>17%	0.011±0.025<br>**42**<br>46%
	CCR2	0.11±0.17<br>60%	0.16±0.16<br>**1.5**<br>79%	1.15±1.91<br>**11**<br>100%	0.19±0.14<br>**1.8**<br>88%	0.009±0.02<br>17%	0.12±0.28<br>**13**<br>58%
	CX3CR1	0.0025±0.006<br>20%	0.029±0.06<br>**11.6**<br>36%	0.083±0.19<br>**33**<br>31%	0.1±0.14<br>**40**<br>50%	0.003±0.007<br>17%	0.05±0.16<br>**15**<br>33%
Pro-oxidant activity	iNOS	0.00004±0.00009<br>20%	0.0006±0.002<br>**14.8**<br>14%	0.0002±0.001<br>**3.9**<br>8%	0.0004±0.001<br>**9.4**<br>13%	0.002±0.003<br>33%	0.0004±0.001<br>**0.2**<br>17%
	CYBB	0.05±0.06<br>80%	0.12±0.08<br>**2.3**<br>93%	0.16±0.11<br>**3.1**<br>92%	0.28±0.33<br>**5.5**<br>88%	0.06±0.06<br>67%	0.15±0.20<br>**2.4**<br>83%
	CYBA	0.04±0.05<br>80%	0.12±0.09<br>**3.0**<br>93%	0.3±0.22<br>**7.3**<br>92%	0.13±0.12<br>**3.2**<br>63%	0.083±0.084<br>50%	0.06±0.09<br>**0.7**<br>58%
Anti-oxidant activity	NRF2	0.036±0.05<br>80%	0.05±0.04<br>**1.4**<br>86%	0.14±0.08<br>**3.9**<br>100%	0.14±0.09<br>**4.0**<br>100%	0.035±0.035<br>83%	0.07±0.05<br>**2.0**<br>92%
	GPX1	0.43±0.70<br>80%	1.39±0.86<br>**3.2**<br>100%	3.61±2.4<br>**8.4**<br>100%	3.5±4.4<br>**8.1**<br>100%	0.36±0.37<br>67%	0.97±0.79<br>**2.7**<br>100%
	HMOX1	0.03±0.05<br>60%	0.09±0.08<br>**3.0**<br>71%	0.29±0.22<br>**9.5**<br>100%	0.27±0.37<br>**8.9**<br>63%	0.026±0.024<br>67%	0.1±0.1<br>**3.8**<br>92%
Extracellular matrix degradation	MMP1	Undetectable	Undetectable	0.0003±0.001<br>n.d.<br>8%	Undetectable	Undetectable	Undetectable
	MMP2	0.15±0.34<br>40%	0.05±0.06<br>**0.3**<br>64%	0.03±0.05<br>**0.2**<br>31%	0.06±0.07<br>**0.4**<br>63%	0.011±0.02<br>33%	0.026±0.06<br>**2.4**<br>46%
	MMP9	0.0016±0.003<br>20%	0.001±0.003<br>**0.6**<br>14%	Undetectable	0.00085±0.002<br>**0.5**<br>13%	Undetectable	0.003±0.007<br>n.d.<br>21%
Pathogen recognition	TLR2	Undetectable	0.03±0.04<br>n.d.<br>50%	0.13±0.2<br>n.d.<br>62%	0.07±0.1<br>n.d.<br>50%	0.033±0.05<br>33%	0.068±0.11<br>**2.1**<br>63%
	TLR3	Undetectable	0.004±0.01<br>n.d.<br>29%	0.003±0.01<br>n.d.<br>31%	0.01±0.03<br>n.d.<br>25%	0.00002±0.00004<br>17%	0.004±0.010<br>**231**<br>29%
	TLR7	Undetectable	0.03±0.05<br>n.d.<br>43%	0.08±0.11<br>n.d.<br>85%	0.12±0.23<br>n.d.<br>75%	0.1±0.12<br>50%	0.053±0.123<br>**0.5**<br>54%
	TLR9	0.001±0.002<br>20%	0.001±0.003<br>**1.0**<br>7%	0.0003±0.001<br>**0.3**<br>8%	0.0004±0.001<br>**0.4**<br>13%	Undetectable	0.003±0.001<br>n.d.<br>21%
