# Supplementary material for: Connecting Immune Cell Infiltration to the Multitasking Microglia Response and TNF Receptor 2 Induction in the Multiple Sclerosis Brain
Source: Front Cell Neurosci. 2020 Jul 7;14:190. doi: 10.3389/fncel.2020.00190 (PMC7359043; doi:10.3389/fncel.2020.00190)
Supplement: Supplementary file 3 [file Image_1.pdf]

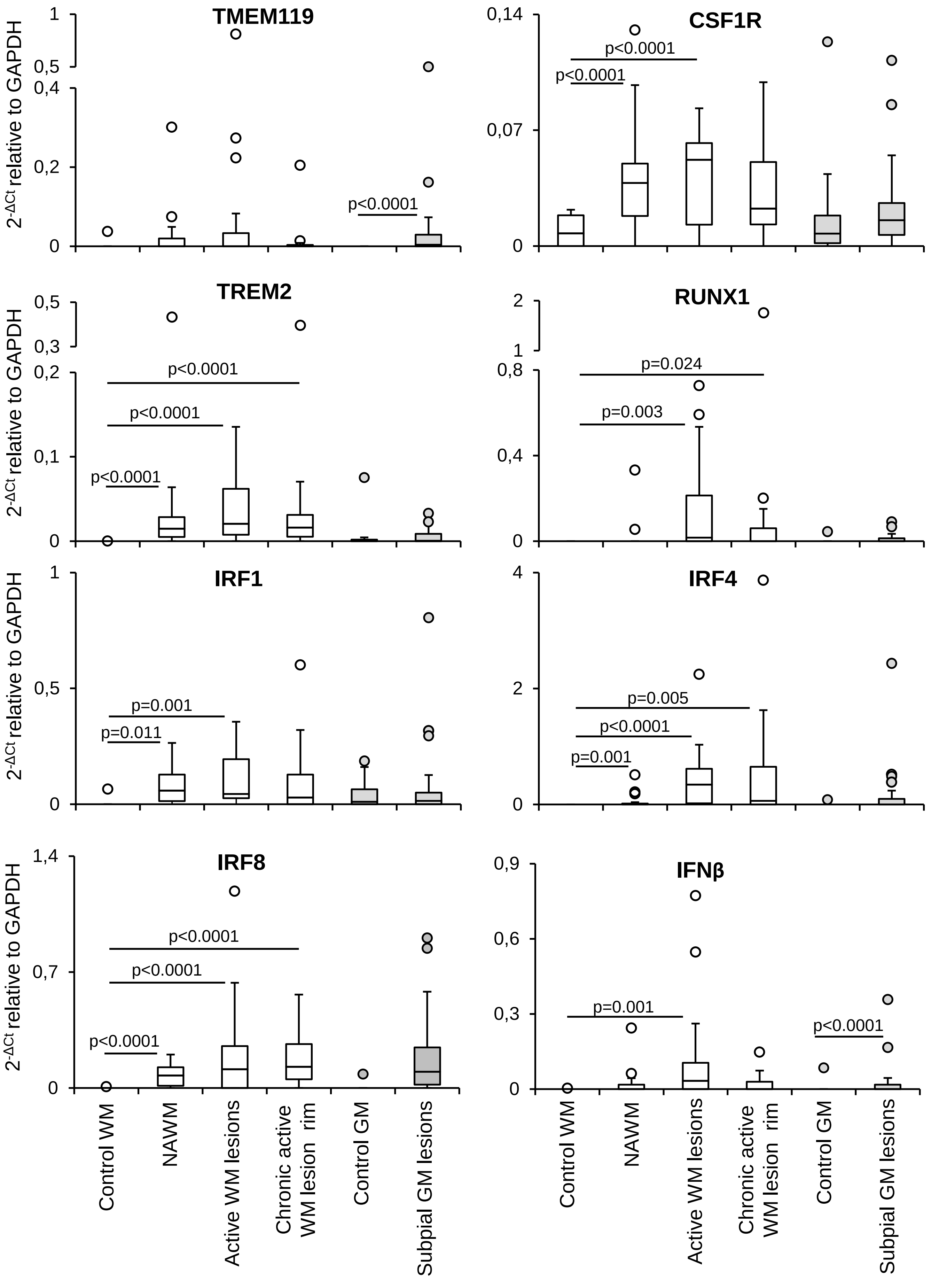
TMEM119
CSF1R
TREM2
RUNX1
IRF1
IRF4
IRF8
IFNβ
$2^{-\Delta Ct}$ relative to GAPDH
p<0.0001
p<0.0001
p<0.0001
p<0.0001
p<0.0001
p<0.0001
p=0.024
p=0.003
p=0.001
p=0.011
p=0.005
p<0.0001
p=0.001
p<0.0001
p<0.0001
p<0.0001
p=0.001
p<0.0001
Control WM
NAWM
Active WM lesions
Chronic active WM lesion rim
Control GM
Subpial GM lesions

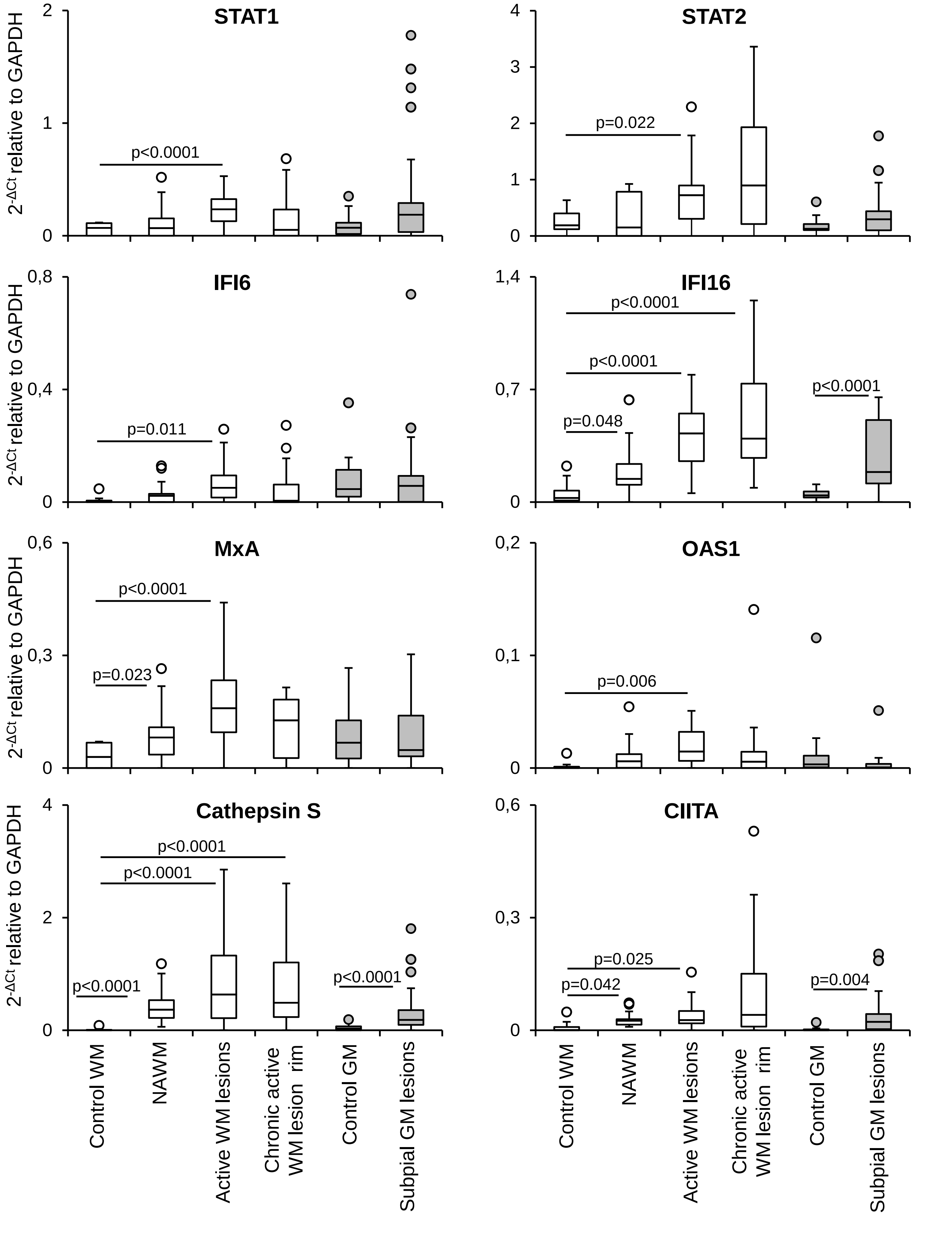
STAT1
STAT2
IFI6
IFI16
MxA
OAS1
Cathepsin S
CIITA
2-ΔCt relative to GAPDH
p<0.0001
p=0.022
p=0.011
p<0.0001
p<0.0001
p=0.048
p<0.0001
p<0.0001
p=0.023
p=0.006
p<0.0001
p<0.0001
p<0.0001
p<0.0001
p=0.025
p=0.042
p=0.004
Control WM
NAWM
Active WM lesions
Chronic active WM lesion rim
Control GM
Subpial GM lesions

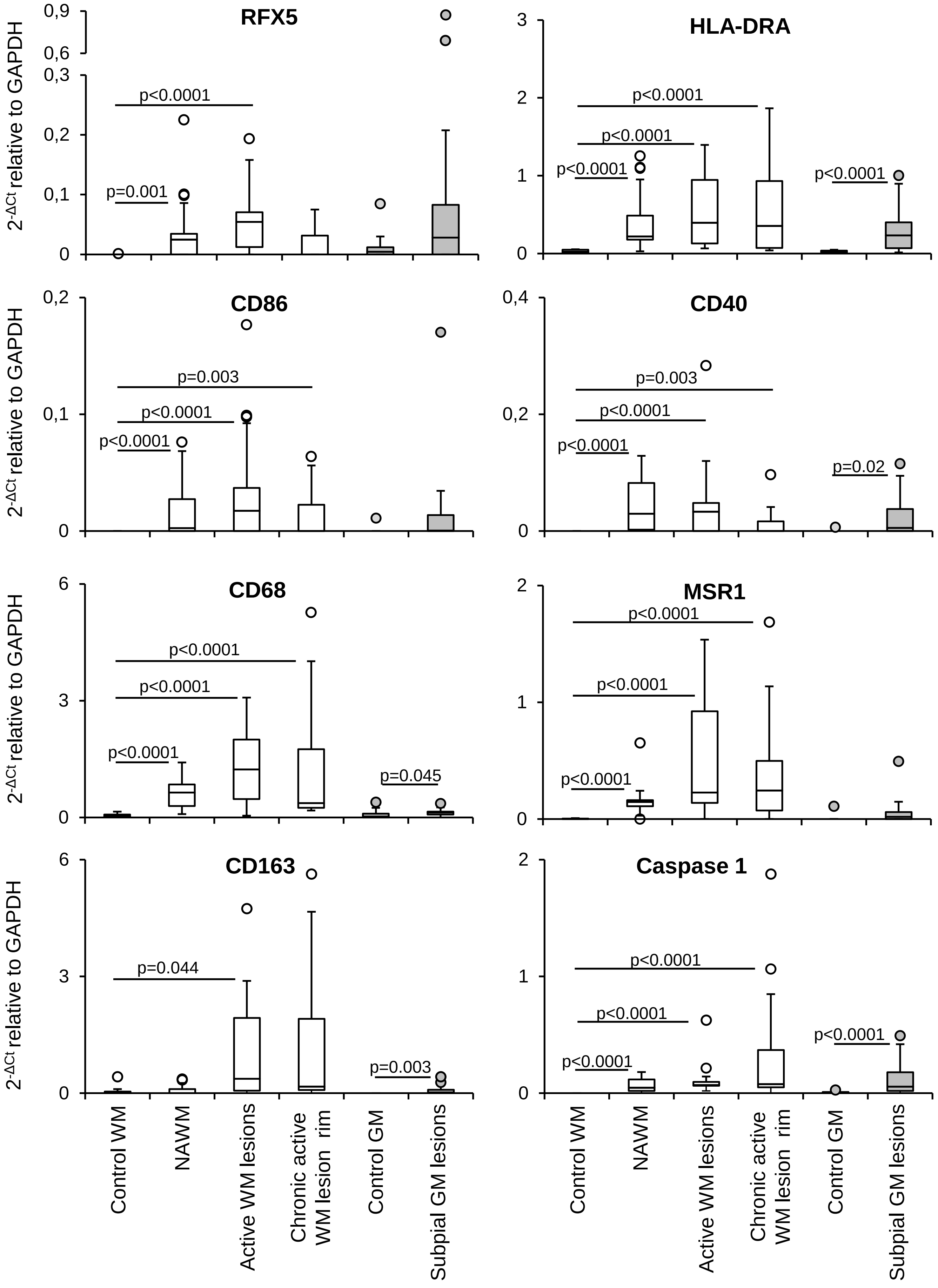

RFX5
HLA-DRA
CD86
CD40
CD68
MSR1
CD163
Caspase 1
2-ΔCt relative to GAPDH
p<0.0001
p=0.001
p=0.003
p=0.02
p=0.045
p=0.044
p=0.003
Control WM
NAWM
Active WM lesions
Chronic active WM lesion rim
Control GM
Subpial GM lesions

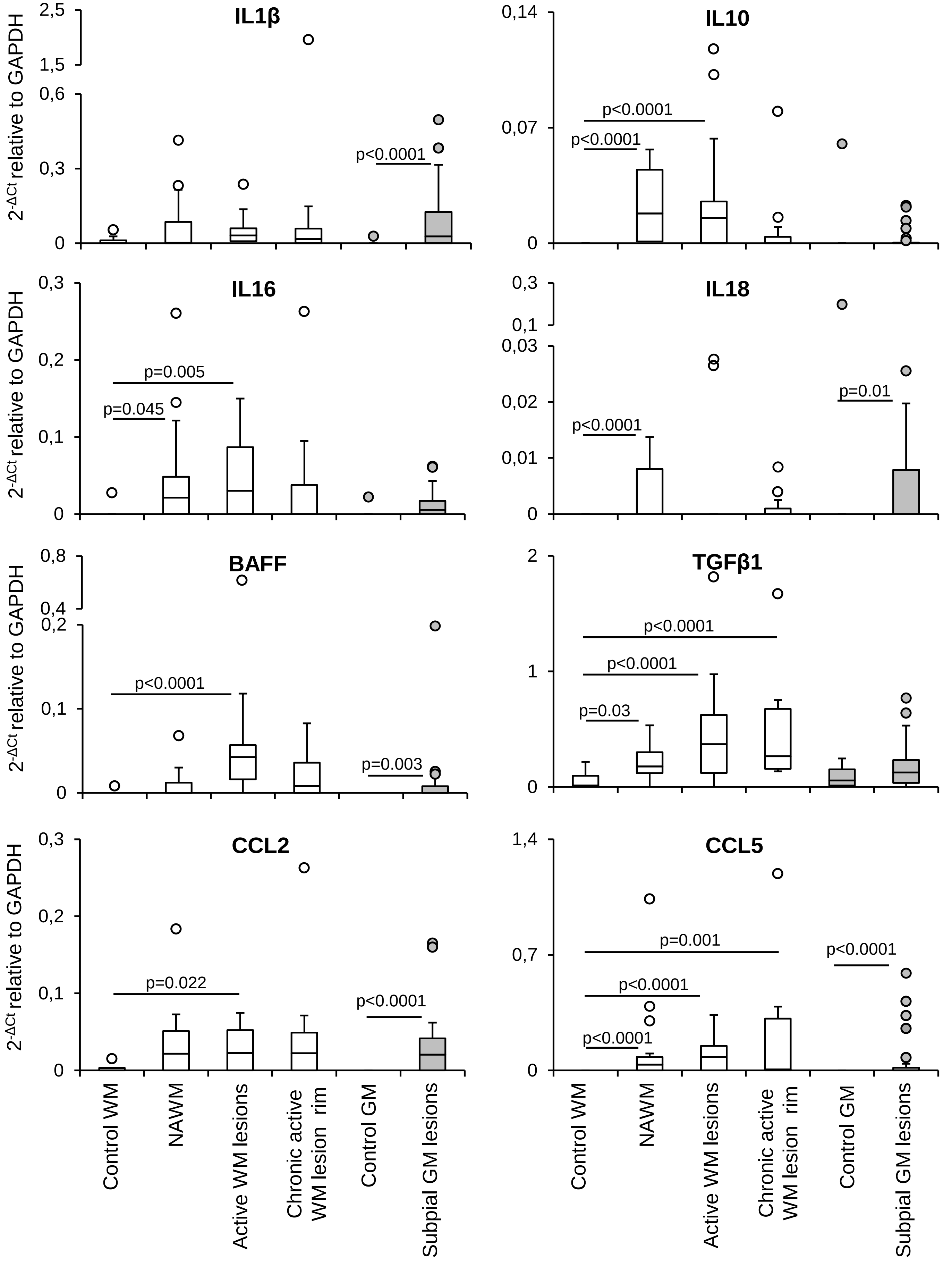

IL1β
IL10
IL16
IL18
BAFF
TGFβ1
CCL2
CCL5
2-ΔCt relative to GAPDH
p<0.0001
p=0.005
p=0.045
p=0.01
p=0.003
p=0.03
p=0.022
p=0.001
Control WM
NAWM
Active WM lesions
Chronic active WM lesion rim
Control GM
Subpial GM lesions

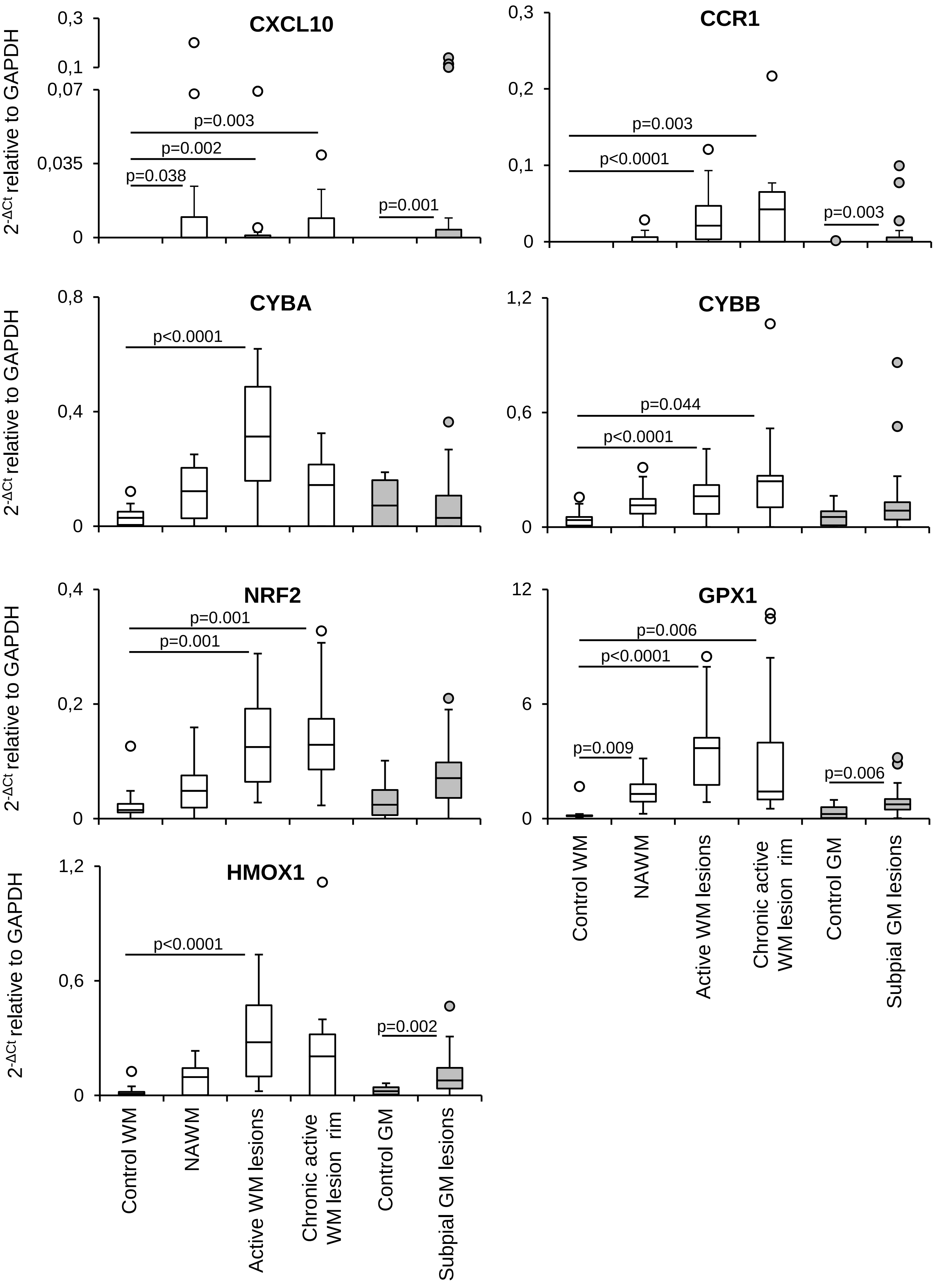
CXCL10
CCR1
CYBA
CYBB
NRF2
GPX1
HMOX1
2-ΔCt relative to GAPDH
p=0.003
p=0.002
p=0.038
p=0.001
p<0.0001
p=0.003
p<0.0001
p=0.044
p=0.001
p=0.001
p=0.006
p<0.0001
p=0.009
p=0.006
p<0.0001
p=0.002
Control WM
NAWM
Active WM lesions
Chronic active WM lesion rim
Control GM
Subpial GM lesions

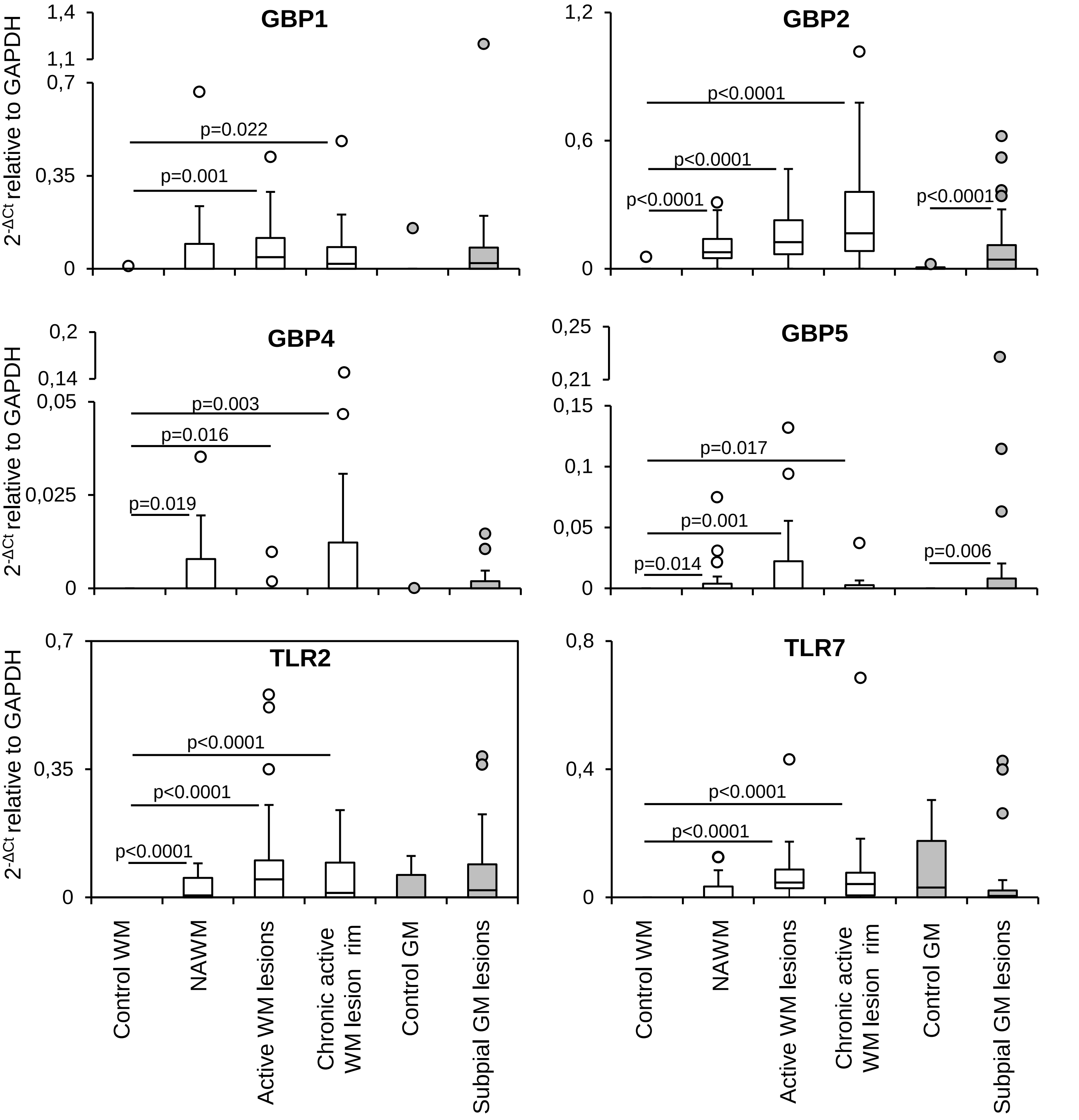

Supplementary Figure 1. Genes differentially expressed in microdissected WM and GM from control and MS brains. The graphs depict the distribution of gene expression values in the indicated control and MS parenchymal areas. Data are expressed as $2^{-\Delta Ct}$ relative to the housekeeping gene GAPDH. Comparisons between control and MS WM and GM areas were performed using the Mann-Whitney test; statistically significant differences ($p < 0.017$ after Bonferroni correction for WM comparisons and $p < 0.05$ for GM comparison) are shown. The lines inside the boxes represent the median value; boxes extend from the 25th to the 75th percentile, covering the interquartile range (IQR), and whiskers extend from the 25th percentile - 1.5 IQR to the 75th percentile + 1.5 IQR. Maximum outliers outside the whiskers are represented by individual marks.
